# Chlorophyll, carotenoid and vitamin C metabolism regulation in *Actinidia chinensis* 'Hongyang' outer pericarp during fruit development

**DOI:** 10.1371/journal.pone.0194835

**Published:** 2018-03-26

**Authors:** Ji-Yu Zhang, De-Lin Pan, Zhan-Hui Jia, Tao Wang, Gang Wang, Zhong-Ren Guo

**Affiliations:** Institute of Botany, Jiangsu Province and Chinese Academy of Sciences, China; NARO Institute of Fruit Tree Science, JAPAN

## Abstract

Ascorbic acid (AsA), chlorophyll and carotenoid contents and their associated gene expression patterns were analysed in *Actinidia chinensis* ‘Hongyang’ outer pericarp. The results showed chlorophyll degradation during fruit development and softening, exposed the yellow carotenoid pigments. *LHCB1* and *CLS1* gene expressions were decreased, while *PPH2* and *PPH3* gene expressions were increased, indicating that downregulation of chlorophyll biosynthesis and upregulation of its degradation, caused chlorophyll degradation. A decrease in the expression of the late carotenoid biosynthesis and maintenance genes (*LCYB1*, *LCYE1*, *CYP1*, *CYP2*, *ZEP1*, *VDE1*, *VDE2*, and *NCED2*) and degradation gene (*CCD1*), showed biosynthesis and degradation of carotenoid could be regulatory factors involved in fruit development. Most genes expression data of L-galactose and recycling pathway were agreement with the AsA concentrations in the fruit, suggesting these are the predominant pathways of AsA biosynthesis. *GMP1*, *GME1* and *GGP1* were identified as the key genes controlling AsA biosynthesis in ‘Hongyang’ outer pericarp.

## Introduction

The genus *Actinidia* (kiwifruit) shows a large variation in flesh colour. These colours are characteristic of the species or specific genotypes and have become an important feature of the fruit. In particular, the yellow- and red-fleshed cultivars have generated great interest throughout the industry worldwide. Previous report showed that the immature fruits of the yellow- and red-fleshed kiwifruit cultivars are green [1]. In contrast to the green-fleshed species, chlorophyll degradation with the unmasking of the carotenoids, occurs in the yellow-fleshed *Actinidia chinensis*, concomitant with the fruit maturation and softening [2].

Chlorophyll metabolism has three distinct phases (**S1 Fig**): synthesis of chlorophyll *a* from glutamate, interconversion between chlorophyll *a* and *b* (chlorophyll cycle), and degradation of chlorophyll *a* into a non-fluorescent chlorophyll catabolites [3, 4]. Some key genes involved in chlorophyll metabolism in kiwifruit are the chlorophyll biosynthesis gene (glutamyl-tRNA reductase, *GluTR*), the light-harvesting chlorophyll *a*/*b* binding complex (*LHCB*), the small subunit of ribulose-1,5-bisphosphatecarboxylase (*RBCS*) and chlorophyll *a* oxygenase (*CAO*), in addition to the chlorophyll degradation genes, chlorophyll *b* reductase (*CBR*), pheophytin pheophorbide hydrolase (*PPH*), pheophorbide *a* oxygenase (*PAO*) and stay-green (*SGR*) [4]. However, other chlorophyll biosynthesis and degradation related genes have not been reported in kiwifruit. Thus, a systematic study of chlorophyll biosynthesis and degradation related genes regulating chlorophyll content is required to understand the chlorophyll degradation mechanism in kiwifruit.

Kiwifruit is rich in carotenoids[5]. *A*. *chinensis* var. *chinensis* (yellow-fleshed) and *A*. *chinensis* var. *deliciosa* (green-fleshed) both contain about 2 μg carotenoids g^-1^ fresh weight, which generally remains stable in the fruit [2]. Carotenoid accumulation is a balance of continual biosynthesis and degradation [6]. Some carotenoid biosynthesis and degradation gene expression changes have been previously examined during kiwifruit maturation and softening (**S2 Fig**). For instance, Ampomah-Dwamena et al. [7] showed that upregulation of ζ-carotene desaturase (*ZDS*) and lycopene β-cyclase (*LCYB*), and downregulation of lycopene ε-cyclase (*LCYE*) and β-carotene hydroxylase (*BCH*), was accompanied by increased carotenoid concentrations, notably β-carotene and lutein, during ripening of *A*. *chinensis* fruit. In particular, *LCYB* plays a significant role in carotenoid accumulation in kiwifruit [7]. The expression levels of carotenoid biosynthesis genes, phytoene synthase (*PSY*), phytoene desaturase (*PDS*) and lycopene cyclases, are the key determinants of carotenoid concentrations in many fruit, such as orange mutant Cara cara (*Citrus*. *sinensis* L. Osbeck) [8], papaya (*Carica papaya* L.) [9], Japanese apricot (*Prunusmume* Siebold & Zucc.) [10], tomato (*Solanum lycopersicum*) [11] and bilberry (*Vaccinium myrtillus* L.) [12]. However, few studies have been undertaken to decipher the carotenoid regulatory mechanisms in kiwifruit.

Kiwifruit is rich in vitamin C (ascorbic acid, AsA), which is one of the key health benefits of the fruit [13]. Four pathways for AsA biosynthesis in plants have been proposed (**S3 Fig**), namely the L-galactose, *myo*-inositol–glucuronate, D-galacturonate, and L-gulose pathways [14]. The L-galactose pathway, which is one of main ascorbate biosynthesis pathways, has been fully documented in various species of higher plants. In *Actinidia* species, most of the genes in this pathway have been identified and cloned [14].

The Chinese cultivar *A*. *chinensis* ‘Hongyang’ is derived from a population grown from seed collected from the wild, in Henan, by the Sichuan Provincial Natural Resources Institute and the Agricultural Bureau, Cangxi County, Sichuan. The fruit skin is thin, green or green-brown. The outer pericarp is light green to yellow-green, the inner pericarp (containing locules) is red, and the core is white [15]. The genomic sequence of this variety has been reported [16] and the chlorophyll, carotenoid and AsA metabolism related genes can be obtained from the Kiwifruit Genome Database (http://bioinfo.bti.cornell.edu/cgi-bin/kiwi/home.cgi).

The current study presents a detailed investigation of *A*. *chinensis* var. *chinensis* ‘Hongyang’ fruit development. AsA, chlorophyll, and carotenoid contents, and their corresponding gene expressions, were monitored during fruit development, to systematically explore the transcription regulatory mechanism of the pigments and AsA metabolism in the outer pericarp of ‘Hongyang’. Simultaneous analysis of various aspects of development including fruit growth, ripening, softening, soluble solids accumulation, titratable acid (TA), soluble sugar content, and organic acids, were also studied.

## Materials and methods

### Plant material and harvest dates

Experiments were carried out using fruit from *A*. *chinensis* ‘Hongyang’ vines, grown at the Institute of Botany, Jiangsu Province and Chinese Academy of Sciences (32°18’ N; 118°52’ E) during 2016. Flowers open at 80% anthesis (22.04.2016) were tagged for subsequent sampling, with three fruit from each of ten vines sampled at weekly intervals, photographed, and tested for firmness and °Brix. For postharvest treatments, fruit from 10 vines was stored in a container at 23 ± 2°C.

### Fruit assessment methods

Following sampling, individual fruits were weighed. The longitudinal, equatorial and lateral diameters of each fruit were measured using a Vernier calliper. Ten fruit were weighed and dried at 65°C for 24 h, to determine fruit dry weight and percentage fruit dry matter. Dry matter was calculated as dry weight/fresh weight × 100. A refractometer (WYT-4, China) was used to determine the soluble solid content (SSC) in juice taken from both ends of the fruit. Fruit firmness was assessed on a 1-mm thick slice of skin and on the outer pericarp at two locations, 90° to the fruit equator, using a Fruit Texture Analyser (GY-4, China), with a 7.9-mm probe, operating at 20 mm s^-1^.

### Biochemical characteristics of fruits

‘Hongyang’ fruit has four distinct tissue types: a central core, an inner pericarp containing locules and seed, a dense outer pericarp, and the skin (**S4 Fig**). At 30 days after anthesis (DAA), the outer pericarp of each fruit was separated, snap frozen in liquid nitrogen and stored at -80°C for later analysis of the soluble sugar content, TA content, AsA content, organic acids contents, chlorophyll and carotenoid contents, and gene expression. The TA was determined by titration of 20 ml of the juice with 0.1 M NaOH and the results expressed as a percentage of citric acid per 100 g juice, according to the Chinese national standard (GB 12293–90). The soluble sugar content was detected using the phenol-vitriolic colourimetric method, with sucrose as the control [17]. The AsA content was measured using high-performance liquid chromatography (HPLC), based on Krupa, Latocha and Liwiñska [18]. Organic acids (oxalic, tartaric, quinic, malic, shikimic, lactic, acetic, citric, fumaric, and succinic acids) were determined as previously described [19]. All the organic acid reagents were of chromatographic grade and provided by Sigma-Aldrich (USA). Chlorophyll and carotenoid contents were analysed using the HPLC method published by Montefiori et al. [2]. All the above-mentioned biochemical characteristics were measured on 10 fruit per repetition.

### Quantitative real-time PCR (qRT-PCR)

The outer pericarp was separated from ten fruit per sample, snap frozen in liquid nitrogen and stored at -80°C. Total RNA was isolated from each kiwifruit sample using the hexadecyltrimethylammonium bromide method [20]. The cDNA was synthesised from the total RNA using the PrimeScript^TM^ RT reagent kit with gDNA Eraser (Perfect Real Time) (TaKaRa, Dalian, China), according to the manufacturer’s instructions.

The chlorophyll biosynthesis and degradation related genes sequences, carotenoid biosynthesis and degradation related genes sequences, and AsA biosynthesis gene sequences, were downloaded from the Kiwifruit Genome Database (http://bioinfo.bti.cornell.edu/cgi-bin/kiwi/home.cgi). Gene primers were designed for each gene using Beacon Designer (**S1 Table**). Kiwifruit *actin* was used as the housekeeping gene to monitor cDNA abundance [21]. The qRT-PCR was performed on an Applied Biosystems 7300 Real-time PCR system with SYBR Premix Ex Taq (Perfect Real Time) (TaKaRa), as described by Zhang et al. [22]. The relative levels of genes to control *actin* mRNAs were analysed using the 7300 PCR system software and the 2^−ΔΔ*C*t^ method [23]. Data were analysed using SPSS (version 17.0, Chicago, IL, USA).

### Statistical analysis

Experimental data were evaluated using analysis of variance (ANOVA). Significant differences among the means of three replicates (*P*< 0.05) were determined by Duncan’s multiple range tests. A Pearson’s correlation coefficient (r) test was carried out on all the qRT-PCR data to find statistically significant correlations between gene expression and total chlorophyll and AsA contents, respectively. The data were analysed on SPSS16.0 for Windows. Heatmap were performed using the software of MEV (Multi Experiment Viewer). Color scale represents log2^−ΔΔ*C*t^ counts where blue indicates low level and red indicates high level.

## Results

### *A*. *chinensis* ‘Hongyang’ fruit development

**Fig 1:**
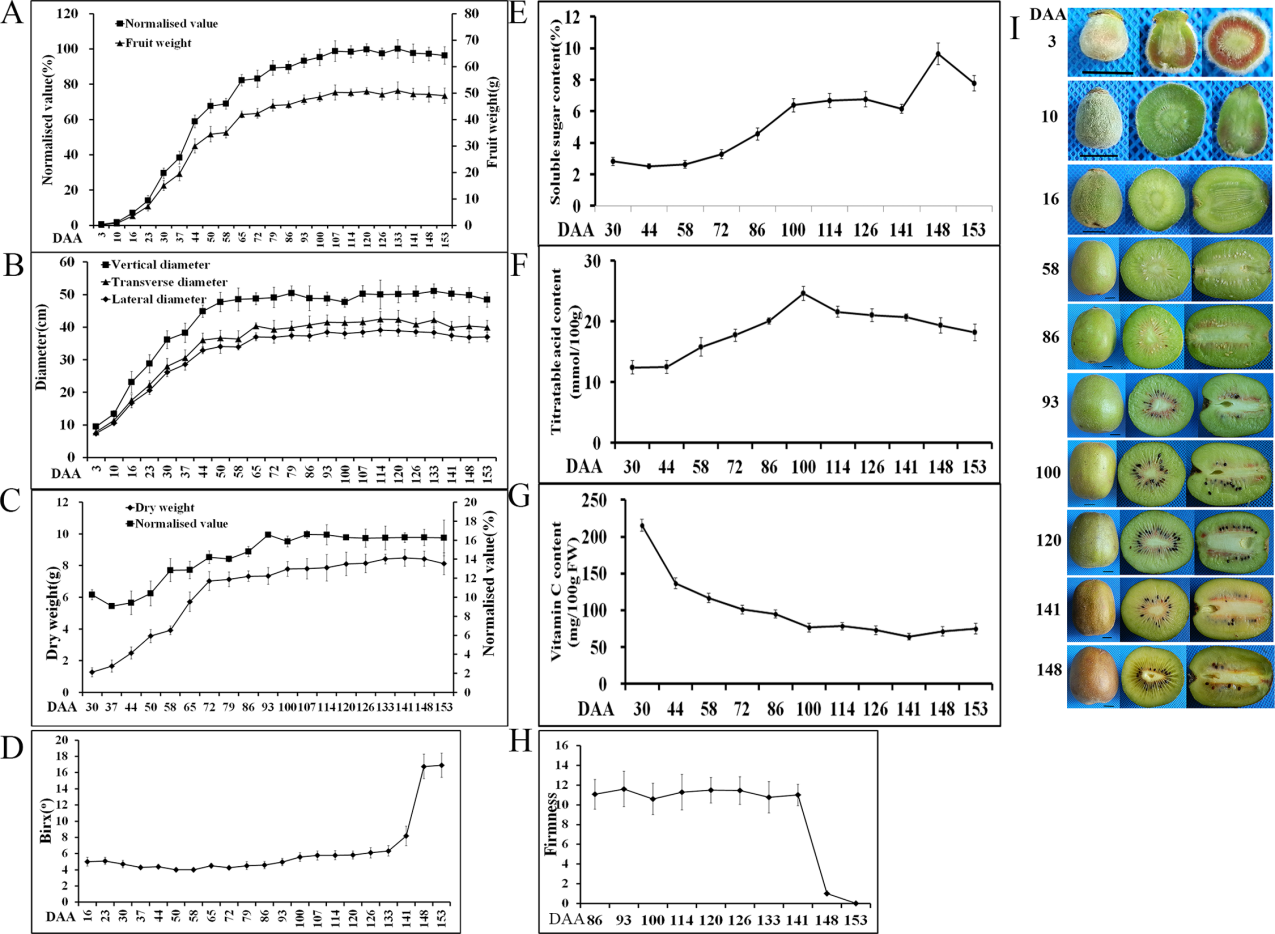
**Development of *A*. *chinensis* var. *chinensis* ‘Hongyang’ from the open flower (0 days after anthesis, DAA) to ripened fruit including fruit weight (A), size (B), dry matter (C), °Brix (D), soluble sugar content (E), titratable acid (F), ascorbic acid (G), firmness (H) and phenotype (I, Bars indicated 1 cm)**. Each value is presented as the mean ± standard deviation (n = 10).

A comprehensive study of *A*. *chinensis* ‘Hongyang’ during fruit development was conducted starting from zero DAA, until the fruit were mature (141 DAA) and ripe (153 DAA). The fruit fresh weight showed a sigmoidal growth curve (**Fig 1A**). After fertilisation, fruit growth increased exponentially, with fruit reaching 14% of their final fresh weight at 23 DAA, and 80% final weight at approximately 65 DAA. Then, the fruit entered a slower growth phase, reaching 98% of the final weight at 107 DAA. Thereafter, the fruit weight remained constant, until 133 DAA, when it decreased slightly as the fruit ripened. The seed began to change colour, initially from white to brown at 86 DAA, finally to black at 93 DAA. Almost all the seeds had turned black at 120 DAA (**Fig 1I**). The mature fruit was harvested at 141 DAA, and stored in a container at 23 ± 2°C. The outer pericarp of the immature ‘Hongyang’ fruit (< 120 DAA) is green. The colour started to change pale green at 141 DAA, and progressed to green—yellow at 148 DAA (7 d after harvest) (**Fig 1I**). Fruit flesh firmness measurement was started at 86 DAA. No changes in flesh firmness were observed from 86–141 DAA but firmness decreased rapidly at 7 d after harvest (**Fig 1H**). There was a significant increase in the SSC in the fruit as the fruit ripening progressed (**Fig 1D**). ‘Hongyang’ fruit has four distinct tissue types: a central core, an inner pericarp containing locules and seed, a dense outer pericarp, and the skin. The inner pericarp colour started to redden at about 86 DAA (**Fig 1I**). The dry weight of the fruit increased linearly between 30–72 DAA (**Fig 1C**). From this point on, the dry weight of the fruit continued to increase, reaching a maximum dry weight at 141 DAA (**Fig 1C**). Comparing the dry weight and fresh weight of the fruit as dry matter percentages, the dry matter content of the fruit was high at 30 DAA, and then decreased, reaching a minimum at 37 DAA. It subsequently increased rapidly until 93 DAA, at which point no further increase in dry matter was observed (**Fig 1C**).

### Changes in soluble sugar, TA and AsA content in the outer pericarp of *A*. *chinensis* ‘Hongyang’ during fruit development

The soluble sugar content increased from 72–100 DAA, and then stabilised. After the fruit was harvested, the soluble sugar content increased rapidly as the fruit ripened and softened, attaining a maximum (9.63%) at 7 d storage, before decreasing (**Fig 1E**). The TA content increased from 44 DAA (12.54%) to 100 DAA (24.61%), and then decreased. After the fruit was harvested, the TA content decreased continually with the fruit ripening and softening (**Fig 1F**). The AsA content was highest at 30 DAA (215.15 mg 100 g^-1^fresh weight) but then declined rapidly until 100 DAA (76.60 mg 100 g^-1^fresh weight), then stabilising (**Fig 1G**).

### Change in organic acid contents in the outer pericarp of ‘Hongyang’ during fruit development

Ten organic acids were detected by HPLC during ‘Hongyang’ fruit development. Eight organic acids were detected but tartaric acid and fumaric acid were not found (**Table 1**). The total organic acids content was 56.90 mg g^-1^ at 58 DAA and maximum at 141 DAA (180.88 mg g^-1^), when the mature fruit was ready for harvesting. After this point, there was a rapid decrease in total organic acids at 148 DAA, at which stage the fruit was soft and edible. Among the organic acid contents in ‘Hongyang’ fruit during development, quinic, malic and citric acids were predominant. The quinic acid content was stable at the early stage of fruit growth (12.81 and 12.88 mg g^-1^ at 58 and 100 DAA, respectively), highest at 141 DAA (19.99mg g^-1^), and then decreased at 148 DAA (13.12 mg g^-1^). The malic acid content was 43.00 mg g^-1^ at 58 DAA, and then decreased at 100 DAA. Malic acid accumulated to the highest level at 141 DAA (153.03 mg g^-1^), as the fruit entered the development period, and then decreased with softening. The citric acid content was lowest at 58 DAA but rapidly accumulated, with the highest level detected at 100 DAA (9.56 mg g^-1^), before it slowly decreased. Shikimic and lactic acid were all detected in the fruit as it entered the development period but the contents were low compared to quinic, malic and citric acids. Oxalic and acetic acids were only detected at the early stage of fruit growth (58 DAA).

**Table 1:** Organic acid composition of *A*. *chinensis* var. *chinensis* ‘Hongyang’ outer pericarp during fruit development.

organic acid composition	DAA (mg/g FW)			
	58	100	141	148
**Oxalic acid**	0.11±0.01	-	-	-
**Tartaric acid**	-	-	-	-
**Quinic acid**	12.81±0.12 a	12.88±0.98 a	19.99±1.04 b	13.12±0.92 a
**Malic acid**	43.00±1.24 c	27.32±0.89 b	153.03±5.90 d	19.35±0.86 a
**Shikimic acid**	0.04±0.00	0.02±0.00	0.01±0.00	0.01±0.00
**Lactic acid**	0.14±0.02 a	0.18±0.02 b	0.14±0.01 a	0.26±0.02 c
**Acetic acid**	0.07±0.00	-	-	-
**Citric acid**	0.70±0.03 a	9.56±0.25 d	7.71±0.37 c	7.11±0.38 b
**Fumaric acid**	-	-	-	-
**Succinic acid**	0.04±0.00	0.07±0.01	-	-
**Total organic acid**	56.90±1.42 b	50.02±2.15 b	180.88±7.31 c	39.86±2.17 a

-: compound not detected. The different small letters for number in a same column represent significant difference at 0.05 level.

### Changes in chlorophyll and total carotenoid in the outer pericarp of ‘Hongyang’ during fruit development

**Fig 2:**
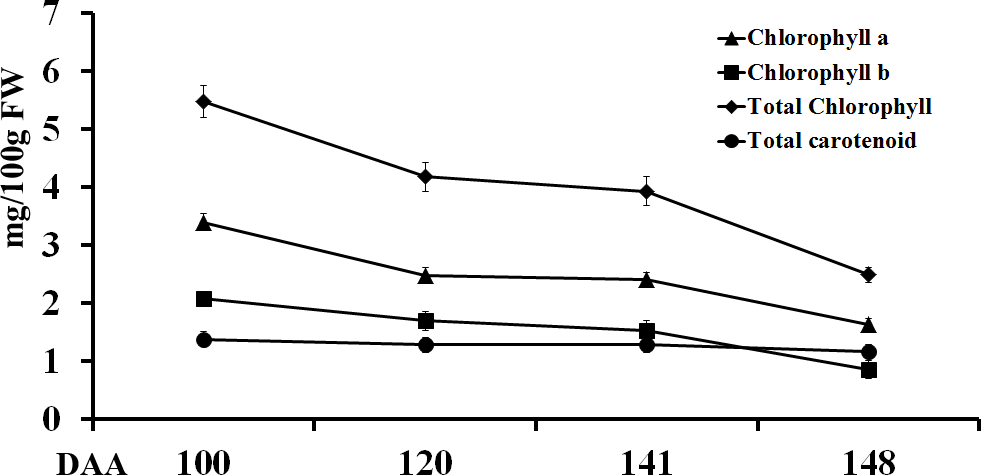
Change in chlorophyll *a*, chlorophyll *b*, total chlorophyll, and total carotenoid contents during *A*. *chinensis* var. *chinensis* ‘Hongyang’ fruit development. Each value is presented as the mean ± standard deviation (n = 10).DAA, days after anthesis.

The outer pericarp of immature ‘Hongyang’ fruit (< 120 DAA) was green. The colour started to change at 141 DAA and progressed to green—yellow at 148 DAA (7 d after harvest) (**Fig 1B**). The concentrations of chlorophyll *a*, chlorophyll *b* and total chlorophyll were decreased with the outer pericarp development stage (**Fig 2** and **S2 Table**). In contrast, there was no marked change in the total carotenoid concentration during fruit development (**Fig 2** and **S2 Table**).

### The expression of chlorophyll biosynthesis and degradation related genes in the outer pericarp of ‘Hongyang’

**Fig 3:**
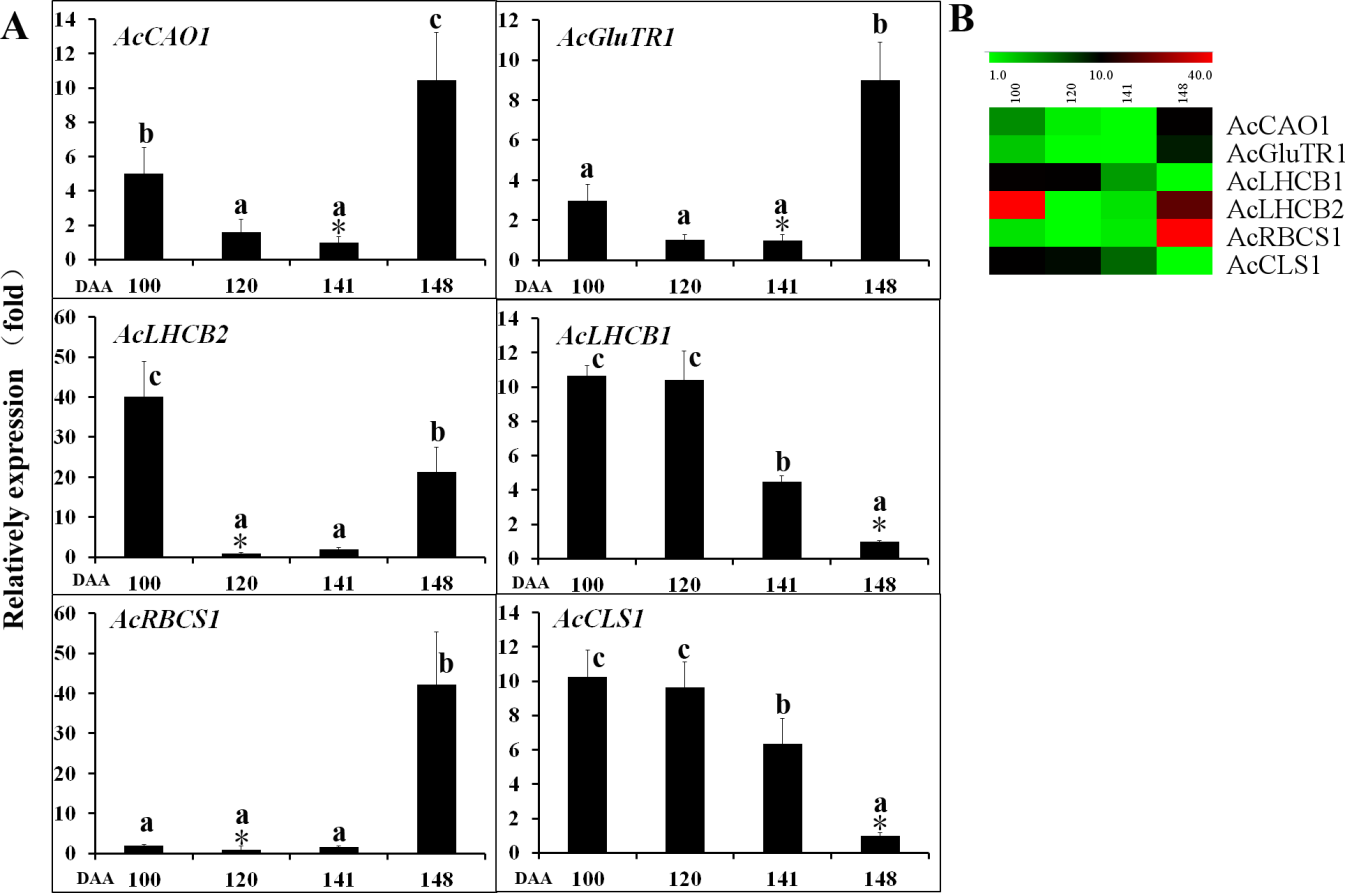
Expression of chlorophyll biosynthetic pathway associated genes in *A*. *chinensis* var. *chinensis* Hongyang’ outer pericarp during fruit development. *CAO1*, chlorophyll *a* oxygenase 1; *GluTR1*, glutamyl-tRNA synthase 1; *RBCS1*, ribulose-1,5-bisphosphate carboxylase/oxygenase small subunit 1; *LHCB1*, light-harvesting chlorophyll *a*/*b* binding complex 1; *LHCB2*, light-harvesting chlorophyll *a*/*b* binding complex 2; *CLS1*, chlorophyll synthase 1. Error bars indicate standard error (n = 3). The asterisk (*) represents that the sample was used as reference in relative comparison. The different small letters for number in a same gene represent significant difference at 0.05 level. Heatmap were performed using the software of MEV (Multi Experiment Viewer). Color scale represents log2^−ΔΔ*C*t^ counts where blue indicates low level and red indicate high level.

The expression of the genes involved in chlorophyll biosynthesis in the outer pericarp of ‘Hongyang’was measured by qRT-PCR (**Fig 3 and S3 Table**). Expression of *LHCB1*and *CLS1* genes were decreased with fruit development and yellowing. A similar pattern was observed for the *CAO1*, *GluTR1* and *LHCB2* genes, with initial fruit maturation but their expression levels increased at 148 DAA, when the fruit was soft and edible. *RBCS1* expression was maximal at 148 DAA.

**Fig 4:**
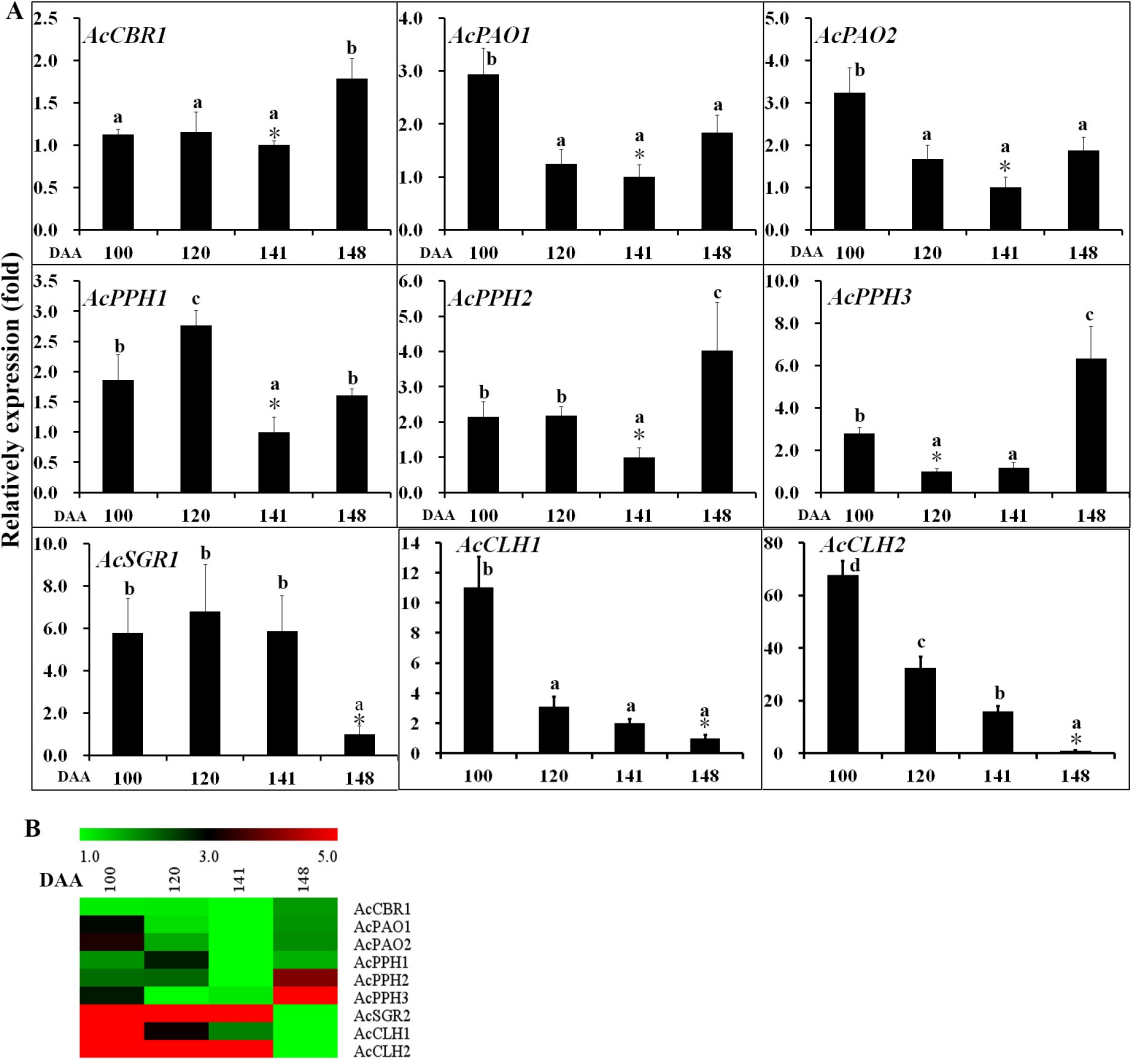
Gene expression of chlorophyll degradation pathway in *A*. *chinensis* var. *chinensis* ‘Hongyang’ outer pericarp during fruit development. *CBR1*, chlorophyll *b* reductase; *PAO1*, pheophorbide *a* oxygenase 1; *PAO2*, pheophorbide *a* oxygenase 2; *PPH1*, pheophytin pheophorbide hydrolase 1; *PPH2*, pheophytin pheophorbide hydrolase 2; *PPH3*, pheophytin pheophorbide hydrolase 3; *SGR1*, stay-green 1; *CLH1*, chlorophyllase 1; *CLH2*, chlorophyllase 2. Error bars indicate standard error (n = 3). The asterisk (*) represents that the sample was used as reference in relative comparison. The different small letters for number in a same gene represent significant difference at 0.05 level. Heatmap were performed using the software of MEV. Color scale represents log2^−ΔΔ*C*t^ counts where blue indicates low level and red indicate high level.

The expression of the chlorophyll degradation genes was also analysed in the outer pericarp of ‘Hongyang’ by qRT-PCR (**Fig 4 and S3 Table**). Expression of *CLH1* and *CLH2* genes were decreased with fruit development and yellowing. *CBR1* expression was slightly increased at 148 DAA. *PAO1* and *PAO2* gene expression levels initially decreased with fruit maturation but increased at 148 DAA. Expression of *PPH2* and *PPH3* were higher at 148 DAA than those at 100, 120, and 141 DAA, Expression of *PPH1* fluctuated slightly during fruit development.

A Pearson’s correlation coefficient (r) test was carried out on all the qRT-PCR data to identify any statistically significant correlations between gene expression and total chlorophyll content (**S4 Table**). There was a significant positive correlation for *CLH2* and *CLS1* expression with total chlorophyll content. No other significant correlations with total chlorophyll content were found.

### The expression of carotenoid biosynthesis and degradation related genes in the outer pericarp of ‘Hongyang’

**Fig 5:**
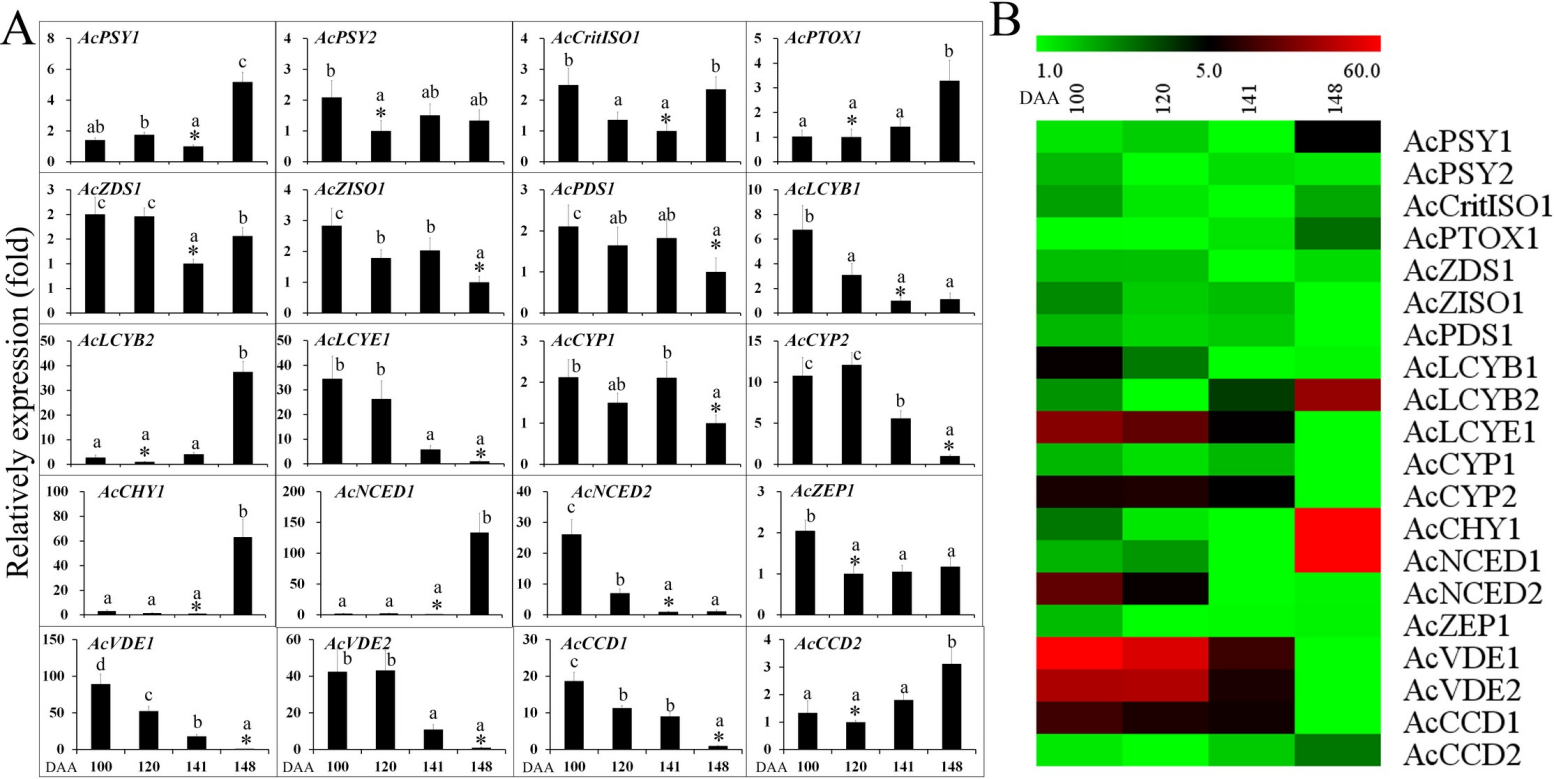
The expression of carotenoid biosynthesis and degradation genes in *A*. *chinensis* var. *chinensis* ‘Hongyang’ outer pericarp during fruit development. *CCD*, carotenoid cleavage dioxygenases; *CHY*, non-heme hydroxylases; *CRTISO*, 7,9,7',9'-tetra-*cis*-lycopene isomerase; *CYP*, P450 hydroxylase; *LCYB*, lycopene β-cyclase; *LCYE*, lycopene γ-cyclase; *NCED*, 9*-cis-*epoxycarotenoid dioxygenase; *PDS*, phytoene desaturase; *PSY*, phytoene synthase; *PTOX*, alternative oxidase; *VDE*, violaxanthin de-epoxidase; *ZDS*, ζ-carotene desaturase; *ZEP*, zeaxanthin epoxidase; *ZISO*, 9,15,9’-tri-*cis-*ζ-carotene isomerase. Error bars indicate standard error (n = 3). The asterisk (*) represents that the sample was used as reference in relative comparison. The different small letters for number in a same gene represent significant difference at 0.05 level. Heatmap was performed using the software of MEV. Color scale represents log2^−ΔΔ*C*t^ counts where blue indicates low level and red indicate high level.

Expression of the carotenoid biosynthesis and degradation related genes were analysed in the outer pericarp of ‘Hongyang’ fruit at four different developmental stages (**Fig 5 and S5 Table**) by qRT-PCR. All the 18 examined biosynthesis genes and the two examined degradation genes were expressed at measurable levels throughout fruit development but with variable expression patterns (**Fig 5 and S5 Table**). The expression of the biosynthesis genes *PSY1*, *PTOX1*, *LCYB2* and *CHY1* showed highly similar patterns, with relatively low expression at the fruit development (100 and 120 DAA) and maturation (141 DAA) stages but revealed an approximate five-, three-, 37- and 63-fold increase, respectively, at the fruit ripening stage (148 DAA). The expressions of the biosynthesis genes *ZISO1*, *PDS1*, *LCYB1*, *LCYE1*, *CYP1*, *CYP2*, *NCED2*, *ZEP1*, *VDE1* and *VDE2* were high at 100 DAA but decreased during fruit development. The expression of *NCED1* increased significantly at the fruit ripening stage. The expression of carotenoid degradation related gene *CCD1* show an evident decrease with fruit development.

### The expression of AsA biosynthesis and recycling pathway genes in the outer pericarp of ‘Hongyang’

**Fig 6:**
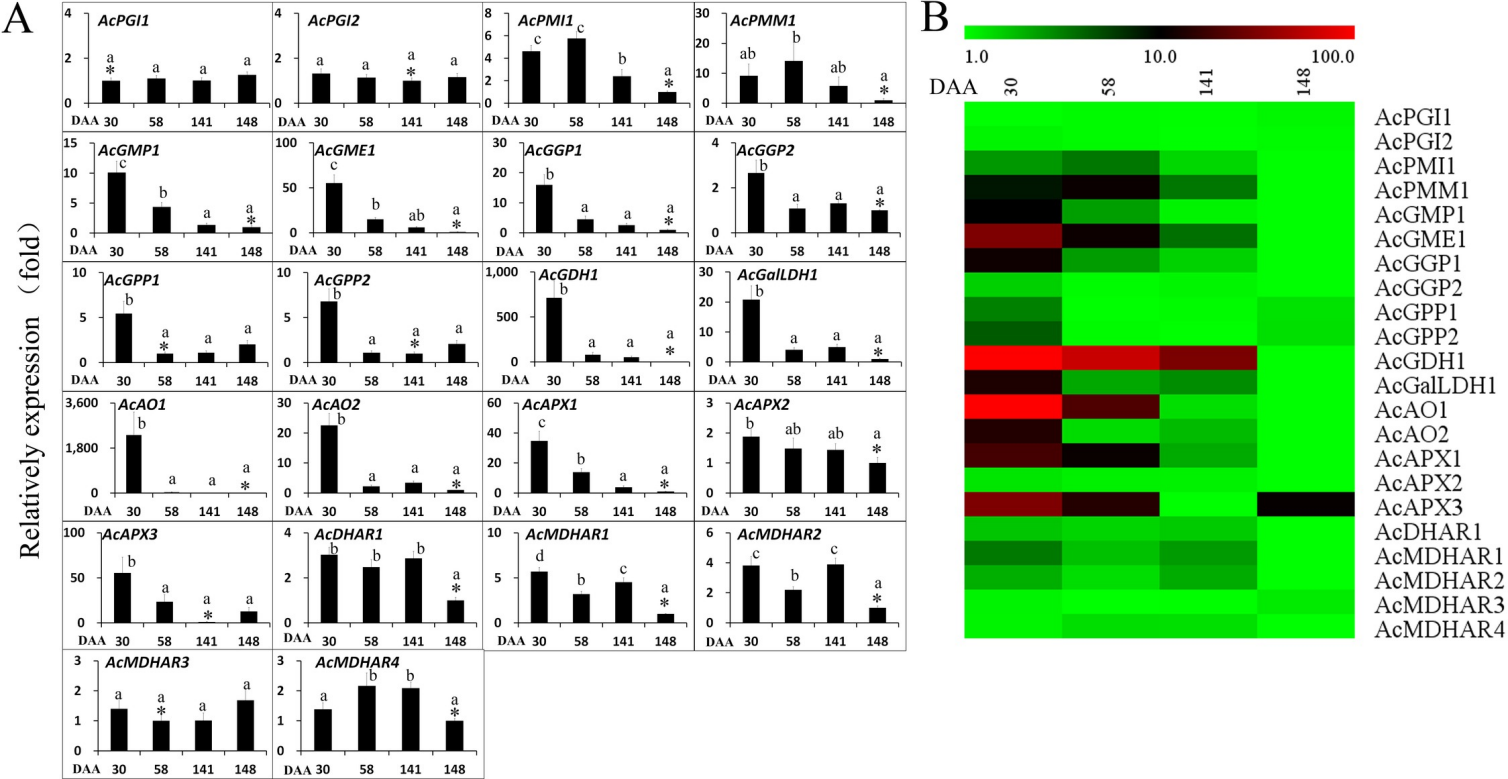
The expression of ascorbic acid biosynthesis and recycling pathway genes in *A*. *chinensis* var. *chinensis* ‘Hongyang’ outer pericarp during fruit development. *AO*, L-ascorbate oxidase; *APX*, L-ascorbate peroxidase; *DHAR*, dehydroascorbate reductase; *GalLDH*, L-galactono-1,4-lactone dehydrogenase; *GDH*, L-galactose dehydrogenase; *GGP*, GDP-L-galactose phosphorylase; *GME*, GDP-D-mannose-3,5-epimerase; *GMP*, GDP-D-mannose pyrophosphorylase; *GPP*, L-galactose-1-phosphate phosphatase; *MDHAR*, monodehydroascorbate reductase; *PGI*, glucose-6-phosphate isomerase; *PME*, pectinesterase; *PMI*, mannose-6-phosphate isomerase; *PMM*, phosphomannomutase. Error bars indicate standard error (n = 3).The asterisk (*) represents that the sample was used as reference in relative comparison. The different small letters for number in a same gene represent significant difference at 0.05 level. Heatmap was performed using the software of MEV. Color scale represents log2^−ΔΔ*C*t^ counts where blue indicates low level and red indicate high level.

The AsA content declined rapidly during fruit development, until 100 DAA, then stabilised (**Fig 2C**). In order to understand the mechanism of AsA biosynthesis in the outer pericarp of ‘Hongyang’, the expressions of AsA biosynthesis and recycling pathway genes were determined using qRT-PCR (**Fig 6 and S6 Table**). There was no marked change in the expression of *PGI1* and *PGI2* during fruit development, whereas the *PMI1* and *PMI2* expression levels were upregulated at 58 DAA, and then decreased significantly as the fruit matured and softened. The expression of the AsA biosynthesis genes including *GMP1*, *GME1*, *GGP1*, *GGP2*, *GPP1*, *GPP2*, *GDH1*, and *GalLDH1*, showed highly similar patterns, with high levels of expression at the early stage of fruit development (30 DAA), before decreasing significantly. The recycling genes *AO1*, *AO2*, and *APX1* had an expression pattern similar to that of the AsA biosynthesis genes *GMP1*, *GME1*, *GGP1*, *GGP2*, *GPP1*, *GPP2*, *GDH1*, and *GalLDH1*. The expressions of *MDHAR1* and *MDHAR2* decreased at 58 DAA, increased at fruit maturation (141 DAA), and then decreased at fruit softening (148 DAA). The expression of *DHAR1* was decreased at fruit softening (148 DAA). *APX3* expression decreased rapidly until fruit maturation but increased slightly at fruit softening. The expression of *MDHAR3* did not change remarkably during fruit development. Statistically significant correlations between gene expression and total AsA content were analysed. The results showed a significant positive correlation for *GMP1*, *GME1*, *GGP1*, *GGP2*, *GPP2*, *GDH1*, *AO*, *APX1* and *APX3* expression with AsA content (**S7 Table**). The expression level relations between each pair of *GMP1*, *GME1*, *GGP1*, *GGP2*, *GPP1*, *GPP2*, *GDH1*, *GalLDH1*, *AO1*, *AO2* and *APX1* were significantly and positively correlated, except for *GMP1* and *GPP1*, *GMP1* and *GPP2*, *APX1* and *GPP1*, and *APX1* and *GPP2* (**S7 Table**).

## Discussion

### Chlorophyll degradation in the outer pericarp of ‘Hongyang’ with fruit development and softening

The chlorophyll, anthocyanin and carotenoid contents determine the flesh colour of kiwifruit. The immature fruits are green in *A*. *deliciosa* and *A*. *chinensis* [1]. Montefiori et al. [2] showed the chemical composition and ultrastructure of *A*. *chinensis* fruit changed with fruit maturation and ripening stages, as chlorophyll was degraded to colourless catabolites, unmasking the yellow carotenoids already present rather than increased carotenoid synthesis. This was supported by Pilkington et al. [4], who reported that the concentration of carotenoids was constant and similar in both *A*. *deliciosa* cv. ‘Hayward’ (green) and *A*. *chinensis* cv. ‘Hort16A’ (gold) fruit types across development, where as the chlorophyll concentration decreased with fruit development. Like *A*. *chinensis* cv. ‘Hort16A’, the outer pericarp of immature ‘Hongyang’ fruit (<120 DAA) is green. The colour started to change at 141 DAA, and progressed to green—yellow at 148 DAA (**Fig 1**). In concurrence with previous reports [2, 4], the carotenoid concentration remained constant during development of the outer pericarp of ‘Hongyang’, whereas the chlorophyll concentration decreased (**Fig 2**), indicating the amount of chlorophyll determine the colour of the outer pericarp of ‘Hongyang’. In contrast, immature fruit of other crop plants, such as pepper, tomato banana and orange, are green and lose chlorophyll upon ripening.

### Chlorophyll biosynthesis and degradation

The chlorophyll content is determined by its biosynthesis and degradation, and is regulated by the genes and the environment [24]. As above-mentioned, there are three stages of chlorophyll metabolism including chlorophyll biosynthesis, cycle, and degradation [4]. Glutamyl-tRNA reductase (*GluTR*) is the rate-controlling point of the chlorophyll biosynthetic pathway [3]. In the current study, *GluTR1* initially decreased, until 141 DAA, indicating that chlorophyll biosynthesis was downregulated during fruit development until maturation. Chlorophyll synthase (CLS) is a key enzyme in the chlorophyll cycle [4]. Expression of the *CLS1* gene decreased during fruit development, suggesting that *CLS1* plays key role in the process of chlorophyll degradation. The *CAO* genes are slightly downregulated in gold kiwifruit across fruit development compared to green kiwifruit, suggesting a coordinated downregulation of the biosynthesis pathway [4]. *CAO* and *GluTR* showed similar expression patterns, suggesting that chlorophyll biosynthesis was downregulated during fruit development until fruit maturation. The light-harvesting chlorophyll *a*/*b* binding (LHCB) complex of photosystem II is integral for photosynthesis, and binds chlorophylls *a* and *b* [25, 26]. The degradation of this complex is tightly regulated during senescence, to prevent photochemical damage to the reaction center [26]. The *LHCB1* and *LHCB2* genes were down regulated with chlorophyll degradation in the outer pericarp of ‘Hongyang’. PPH, a key enzyme in the chlorophyll degradation pathway, is an *a*/*b* hydrolase localised in the chloroplast and regulated during senescence [27]. Pilkington et al. [4] stated that the chlorophyll degradation *PPH* gene was similarly expressed in green and gold kiwifruit, indicating that the differences in flesh colour between *A*. *deliciosa* and *A*. *chinensis* were not due to lack of *PPH* expression. In the current study, however, expression level of *PPH2* and *PPH3* was higher at 148 DAA than at 100, 120, and 141 DAA, demonstrating that *PPH2* and *PPH3* participate in chlorophyll degradation during fruit softening. These results suggested that down regulation of chlorophyll biosynthesis and upregulation of chlorophyll degradation result in chlorophyll degradation in the outer pericarp of ‘Hongyang’.

### Carotenoid content during fruit development in the outer pericarp of ‘Hongyang’

The carotenoid content shows a decreasing trend in some fruits during the fruit ripening, such as strawberry [28], raspberries [29], grape [30], apple [31] and bilberry [12], while in the same fruit, accumulation of the anthocyanin content results in red pigment formation. However, in some other fruit, such as citrus [8] and red-fleshed watermelon (*Citrullus lanatus*) [32], the carotenoid content increases during fruit maturation. In contrast, ‘Hongyang’ kiwifruit showed a stable trend in the total carotenoid content during fruit development (**Fig 2**).

Lycopene is a precursor of β-carotene. There was no marked change in the expression of early biosynthesis genes (*PSY2*, *ZDS1*, *CRTISO1*) in the outer pericarp of ‘Hongyang’ fruit during fruit development, indicating the biosynthesis of lycopene was stable. Expression of the late biosynthesis genes (*LCYB1*, *LCYE1*, *CYP1*, *CYP2*, *ZEP1*, *VDE1*, *VDE2* and *NCED2*) and the degradation related gene (*CCD1*) decreased during fruit development, showing that the biosynthesis and degradation of carotenoids were tightly regulated. Thus, the unchanged total carotenoid content throughout the fruit development coincided with the balance in the expression of biosynthesis and degradation related genes at the fruit ripening. However, the mechanisms of carotenoid biosynthesis are different in various species. For instance, Karppinen et al. [12] detected a decrease in the levels of all carotenoid compounds during the fruit development, which does not coincide with the notable increase in the expression of the carotenoid biosynthesis genes at bilberry fruit ripening.

Key carotenoid biosynthetic genes have been isolated and used to examine gene expression during ripening in more than one species of kiwifruit [7]. The concentrations of carotenoids, notably β-carotene and lutein, increase during ripening of *A*. *chinensis* var. *chinensis* fruit, and this is accompanied by increased transcript amounts for zeta-carotene desaturase and LCYβ. Carotenoid accumulation is a balance of continual biosynthesis and degradation. The result of this interplay is important for the colour resulting from chromoplasts in fruit and flowers. *CCD* gene mutation in *Chrysanthemum* [33] and *Brassica* [34] could help generate yellow flower colour, and in peach cultivars [35, 36] and papayas [37]could generated white- orpale-fleshed fruit.

### Key genes controlling AsA biosynthesis in the outer pericarp of ‘Hongyang’

The concentration and accumulation rates of fruit ascorbate vary markedly among fruit species, providing an excellent model to investigate gene factors that control AsA [38]. AsA content was highest at 30 DAA (215.15 mg 100 g^-1^fresh weight), declined rapidly until 100 DAA, and then stabilised (**Fig 1G**), thus, resembling the pattern of *A*. *deliciosa* ‘Hayward’ and *A*. *eriantha* [38]. In order to understand the AsA regulatory mechanisms, the L-galactose pathway and recycling pathway related genes were investigated.

The expression data of most of the L-galactose and recycling pathway genes were in agreement with the AsA concentration in the fruit. Also, there was a significant positive correlation for *GMP1*, *GME1*, *GGP1*, *GGP2*, *GPP2* and *GDH1* expression with AsA content, suggesting that the L-galactose pathway and recycling pathway are the predominant routes of AsA biosynthesisin the outer pericarpof ‘Hongyang’. L-Galactose and L-gulose pathways are the predominant routes to AsA in plants [39], and the L-galactose pathway is the predominant routes of AsA biosynthesis in kiwifruit [38]. AsA biosynthesis through L-galactose pathway supplemented by AsA recycling collectively contributed to accumulating and remaining higher AsA level in kiwifruit cv. ‘White’ during postharvest [40]. Moreover, GalDH activity and relative expressions of the genes *GMP*, *GPP*, *GGP*, *GalDH* and *GalUR* genes were important for regulation of AsA biosynthesis, and the activity and expression of *DHAR* were primarily responsible for regulation of AsA recycling in kiwifruit ‘White’ during postharvest [40].

AsA biosynthesis during early fruit development is the main factor for its accumulation in kiwifruits. The level of *GME* transcripts was not highly correlated with AsA content and rates of accumulation during fruit development in *A*. *deliciosa* (Qinmei). *GMP* transcript levels showed a certain correlation with AsA content and accumulation rate during kiwifruit development. *GPP* is a good candidate for regulating AsA biosynthesis whereas GDP-L-galactose-1-phosphate phosphorylase is not [41]. Previous studies showed that *GGP* is an important regulator of AsA biosynthesis in many plants [42–46]. Thus, it could be assumed that *GGP* controls ascorbate biosynthesis. Bulley et al. [42] studied the transient and stable transformation of *A*. *thaliana* and *Nicotiana benthamiana* with kiwifruit genes and concluded that *GGP* and *GME* synergistically control AsA biosynthesis. In different kiwifruit species, expression of *GGP* and *GME* genes relates to the AsA concentrations with different ascorbate concentrations [42, 45]. Overexpression of kiwifruit or *Arabidopsis GGP* in strawberry, potato and tomato have been shown to significantly increase AsA [34]. These results showed that transcriptional regulation of the key genes *GGP* and *GME* in the L-galactose pathway controls AsA concentrations. A significant positive correlation was noted between the relative *GMP1*, *GME1* or *GGP1* expressions and AsA content. A significant positive correlation was also found between the expression level of each pair of *GMP1*, *GME1* and *GGP1*, which suggested that these three genes control AsA biosynthesis in the outer pericarp of ‘Hongyang’.

## Conclusions

The vitamin C, chlorophyll and carotenoid contents, in addition to their corresponding gene expression patterns, were analysed in the outer pericarp of *A*. *chinensis* ‘Hongyang’. The concentration of chlorophyll *a*, chlorophyll *b* and total chlorophyll decreased with fruit development but the concentration of total carotenoids did not markedly change, confirming that chlorophyll is degraded during fruit development and softening, leaving the yellow pigment of the carotenoids visible. The expression of chlorophyll biosynthesis—associated genes (*LHCB1* and *CLS1*) decreased and the chlorophyll degradation genes (*PPH2* and *PPH3*) were higher at 148 DAA than at 100, 120, and 141 DAA, indicating that downregulation of chlorophyll biosynthesis and upregulation of chlorophyll degradation results in chlorophyll degradation. Expression of the late carotenoid biosynthesis genes (*LCYB1*, *LCYE1*, *CYP1*, *CYP2*, *ZEP1*, *VDE1*, *VDE2* and *NCED2*) and degradation related gene *CCD1* decreased during fruit development, balancing the biosynthesis and degradation of the carotenoids. AsA content was highest at 30 DAA and declined rapidly until 100 DAA during fruit development, before stabilising. The gene expression data of most of the L-galactose and recycling pathway members coincided with the AsA concentration in the fruit, and there was a significant positive correlation between *GMP1*, *GME1*, *GGP1* and *GDH1* expressions with AsA content, suggesting that the L-galactose pathway and recycling pathway are the predominant routes in AsA biosynthesis. A significant positive correlation between the expression levels of each pair among *GMP1*, *GME1* and *GGP1*, suggested that these are the key genes controlling AsA biosynthesis in the outer pericarp of ‘Hongyang’.

## Supporting information

S1 Fig Chlorophyll biosynthesis (white areas) and degradation (gray areas) pathway. GSA: L-glutamic acid-1-semialdehyde; ALA: δ-aminolevulinic acid; PBG: porphobilinogen; Urogen III: Uroprophyrinogen III; Coprogen III: Coproporphyrinogen III; Protogen: Protoporphyrinogen IX; Proto: Protoporphyrin IX; Mg-PPIX: Mg-protoporphyrin IX; Mg-PPIX-ME: Mg-protoporphyrin IX monomethyl eater; Pchlide a: Protochlorophyllide; Chlide a: chlorophyllide a; Chl a: Chlorophyll a; Chl b: Chlorophyll b; Pheide a: Pheophorbide a; Phetin a: Pheophytin a; Enzyme abbreviations: GluTR: GlutamyltRNA reductase; GSA-AT: Glutamate-1-semialdehyde aminotransferase; ALAD: ALA dehydratase; PBGD: Porphobilinogen deaminase; UroS: Uroporphyrinogen III synthase; UroD: Uroporphyrinogen III decarboxylase; CPO: Coproporphyrinogen oxidase; PPXI: Protoporphyrinogen oxidase; MgCh: Mg-chelatase; MTF: Mg-protoporphyrin IX methyltransferase; MTC: Mg-protoporphyrin IX monomethylester cyclase; VR: 8-vinyl reductase; POR: NADPH-protochlorophyllide oxidoreductase; CS: Chlorophyll synthase; CAO: Chlorophyll a oxygenase; CBR: Chlorophyll b reductase; HCR: Hydroxychlorophyll a reductase; Chlase: Chlorophyllase; MCS: Mg-dechelatase; PAO: Pheophorbide a oxygenase; RCCR: Red chlorophyll catabolite reductase.
(DOCX)

S2 Fig Carotenoid biosynthetic pathway in higher plants. DMAPP: 3,3-dimethylpropylene pyrophosphoric acid; IPP: isopentenyl diphosphate; GGPP: Geranylgeranyl pyrophosphate; ABA: Abscisic acid; Enzyme abbreviations: HDR: Hydroxymethylbutenyl -4- phosphate reductase; IPI: IPP isomerase; GGPS: Geranylgeranylpyrophosphate synthase; PSY: Phytoene synthase; PDS: Phytoenedesaturase; ζCDS: ζ-carotene desaturase; CRITSO: Carotenoid isomerase; LCYβ: lycopeneβ-cyclase; LCYε:Lycopene ε-cyclase; βOH: β-ring carotene hydroxylase;εOH: ε-ring carotene hydroxylase; VDE: Violaxanthinde-epoxidase; ZEP: Zeaxanthin epoxidase; CCD: carotenoid cleavage dioxygenases; CCS: Capsanthin synthase; NXS: Neoxanthinsynthase; NCED: Carotenoid cleavage enzymes; ABA2: Short-chain dehydrogenase/redutase; AAO3: Abscisic aldehyde oxidase.
(DOC)

S3 Fig Possible schemes for ascorbate accumulation in fruits. The pathway that uses GDP-L-gulose as a precursor has not been elucidated (Dotted line). AO, L-ascorbate oxidase; APX, L-ascorbate peroxidase; GPI, Glucose-6-phosphate isomerase; PMI, Phosphomannose isomerase; PMM, Phosphomannomutase; GMP, GDP-mannose pyrophosphorylase; GME, GDP-mannose-3,5-epimerase; GGP, GDP-L-galactose-1-phosphate phosphorylase; GPP, L-galactose-1-phosphate phosphatase; GalDH, L-galactose dehydrogenase; GLDH, L-galactono-1,4-lactone dehydrogenase; MDHAR: Monodehydroascorbate reductase; DHAR: Dehydroascorbate reductase.
(DOC)

S4 Fig Tissue of *A*. *chinensis* var. *chinensis* ‘Hongyang’ fruit. Core tissue (pink), inner pericarp (green), outer pericarp (yellow), epidermis (blue).
(DOC)

S1 Table Primers used in qRT-PCR analysis of *A*. *chinensis* var. *chinensis* ‘Hongyang’ outer pericarp. (DOCX)

S2 Table Total carotenoid and total chlorophyll levels in *A*. *chinensis* var. *chinensis* ‘Hongyang’ (mg 100 g^-1^ fresh weight). (DOC)

S3 Table The relative expression (fold) of chlorophyll biosynthesis and degradation related. The different small letters for number in a same gene represent significant difference at 0.05 level.
(DOCX)

S4 Table Pearson’s correlation (r) comparing relative gene expression during *A*. *chinensis* var. *chinensis* ‘Hongyang’ fruit development with total chlorophyll content. **Correlation is significant at the *P* < 0.01 level (1-tailed), *correlation is significant at the *P*< 0.05 level (1-tailed).
(DOC)

S5 Table The relative expression (fold) of carotenoidbiosynthesis and degradation related genes. The different small letters for number in a same gene represent significant difference at 0.05 level.
(DOCX)

S6 Table The relative expression (fold) of AsA biosynthesis and recycling pathway genes. The different small letters for number in a same gene represent significant difference at 0.05 level.
(DOCX)

S7 Table Pearson’s correlation (r) comparing relative gene expression during *A*. *chinensis* var. *chinensis* ‘Hongyang’ fruit development with AsA content. **Correlation is significant at the *P* < 0.01 level (1-tailed), *correlation is significant at the *P*< 0.05 level (1-tailed).
(DOC)
