# Supplementary material for: Chlorophyll, carotenoid and vitamin C metabolism regulation in Actinidia chinensis 'Hongyang' outer pericarp during fruit development
Source: PLoS One. 2018 Mar 26;13(3):e0194835. doi: 10.1371/journal.pone.0194835 (PMC5868826; doi:10.1371/journal.pone.0194835)
Supplement: S1 Fig — GSA: L-glutamic acid-1-semialdehyde; ALA: δ-aminolevulinic acid; PBG: porphobilinogen; Urogen III: Uroprophyrinogen III; Coprogen III: Coproporphyrinogen III; Protogen: Protoporphyrinogen IX; Proto: Protoporphyrin IX; Mg-PPIX: Mg-protoporphyrin IX; Mg-PPIX-ME: Mg-protoporphyrin IX monomethyl eater; Pchlide a: Protochlorophyllide; Chlide a: chlorophyllide a; Chl a: Chlorophyll a; Chl b: Chlorophyll b; Pheide a: Pheophorbide a; Phetin a: Pheophytin a; Enzyme abbreviations: GluTR: GlutamyltRNA reductase; GSA-AT: Glutamate-1-semialdehyde aminotransferase; ALAD: ALA dehydratase; PBGD: Porphobilinogen deaminase; UroS: Uroporphyrinogen III synthase; UroD: Uroporphyrinogen III decarboxylase; CPO: Coproporphyrinogen oxidase; PPXI: Protoporphyrinogen oxidase; MgCh: Mg-chelatase; MTF: Mg-protoporphyrin IX methyltransferase; MTC: Mg-protoporphyrin IX monomethylester cyclase; VR: 8-vinyl reductase; POR: NADPH-protochlorophyllide oxidoreductase; CS: Chlorophyll synthase; CAO: Chlorophyll a oxygenase; CBR: Chlorophyll b reductase; HCR: Hydroxychlorophyll a reductase; Chlase: Chlorophyllase; MCS: Mg-dechelatase; PAO: Pheophorbide a oxygenase; RCCR: Red chlorophyll catabolite reductase. (DOCX) [file pone.0194835.s001.docx]

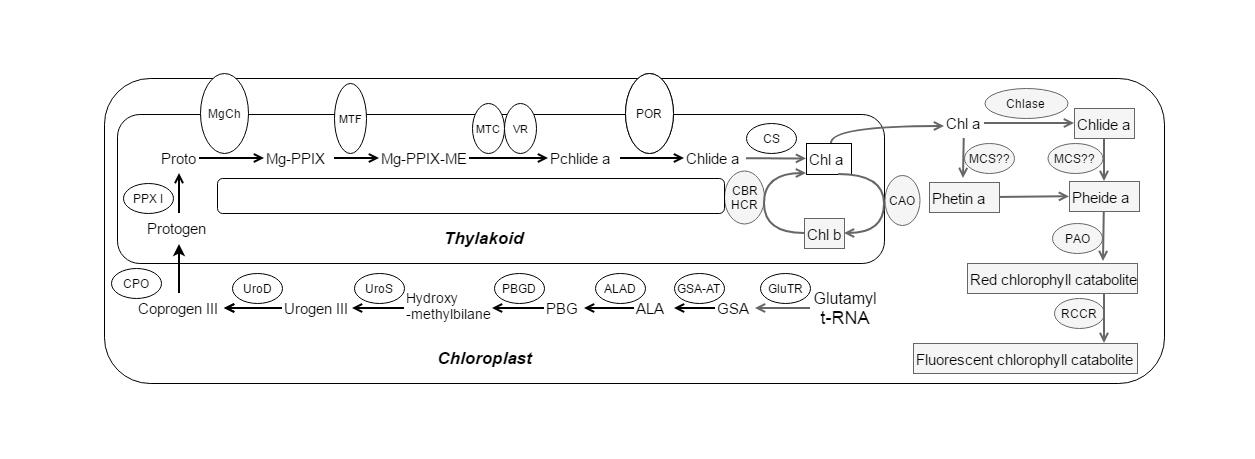


**S1 Fig**. **Chlorophyll biosynthesis (white areas) and degradation (gray areas) pathway.** GSA: L-glutamic acid-1-semialdehyde; ALA: δ-aminolevulinic acid; PBG: porphobilinogen; Urogen III: Uroprophyrinogen III; Coprogen III: Coproporphyrinogen III; Protogen: Protoporphyrinogen IX; Proto: Protoporphyrin IX; Mg-PPIX: Mg-protoporphyrin IX; Mg-PPIX-ME: Mg-protoporphyrin IX monomethyl eater;Pchlide a: Protochlorophyllide; Chlide a: chlorophyllide a; Chl a: Chlorophyll a; Chl b: Chlorophyll b; Pheide a: Pheophorbide a; Phetin a: Pheophytin a; Enzyme abbreviations: GluTR: GlutamyltRNA reductase; GSA-AT: Glutamate-1-semialdehyde aminotransferase; ALAD: ALA dehydratase; PBGD: Porphobilinogen deaminase; UroS: Uroporphyrinogen III synthase; UroD: Uroporphyrinogen III decarboxylase; CPO: Coproporphyrinogen oxidase; PPXI: Protoporphyrinogen oxidase; MgCh: Mg-chelatase; MTF: Mg-protoporphyrin IX methyltransferase; MTC: Mg-protoporphyrin IX monomethylester cyclase; VR: 8-vinyl reductase; POR: NADPH-protochlorophyllide oxidoreductase; CS: Chlorophyll synthase; CAO: Chlorophyll a oxygenase; CBR: Chlorophyll b reductase; HCR: Hydroxychlorophyll a reductase; Chlase: Chlorophyllase; MCS: Mg-dechelatase; PAO: Pheophorbide a oxygenase; RCCR: Red chlorophyll catabolite reductase.
