# Supplementary material for: Chlorophyll, carotenoid and vitamin C metabolism regulation in Actinidia chinensis 'Hongyang' outer pericarp during fruit development
Source: PLoS One. 2018 Mar 26;13(3):e0194835. doi: 10.1371/journal.pone.0194835 (PMC5868826; doi:10.1371/journal.pone.0194835)
Supplement: S2 Fig — DMAPP: 3,3-dimethylpropylene pyrophosphoric acid; IPP: isopentenyl diphosphate; GGPP: Geranylgeranyl pyrophosphate; ABA: Abscisic acid; Enzyme abbreviations: HDR: Hydroxymethylbutenyl -4- phosphate reductase; IPI: IPP isomerase; GGPS: Geranylgeranylpyrophosphate synthase; PSY: Phytoene synthase; PDS: Phytoenedesaturase; ζCDS: ζ-carotene desaturase; CRITSO: Carotenoid isomerase; LCYβ: lycopeneβ-cyclase; LCYε:Lycopene ε-cyclase; βOH: β-ring carotene hydroxylase;εOH: ε-ring carotene hydroxylase; VDE: Violaxanthinde-epoxidase; ZEP: Zeaxanthin epoxidase; CCD: carotenoid cleavage dioxygenases; CCS: Capsanthin synthase; NXS: Neoxanthinsynthase; NCED: Carotenoid cleavage enzymes; ABA2: Short-chain dehydrogenase/redutase; AAO3: Abscisic aldehyde oxidase. (DOC) [file pone.0194835.s002.doc]

**
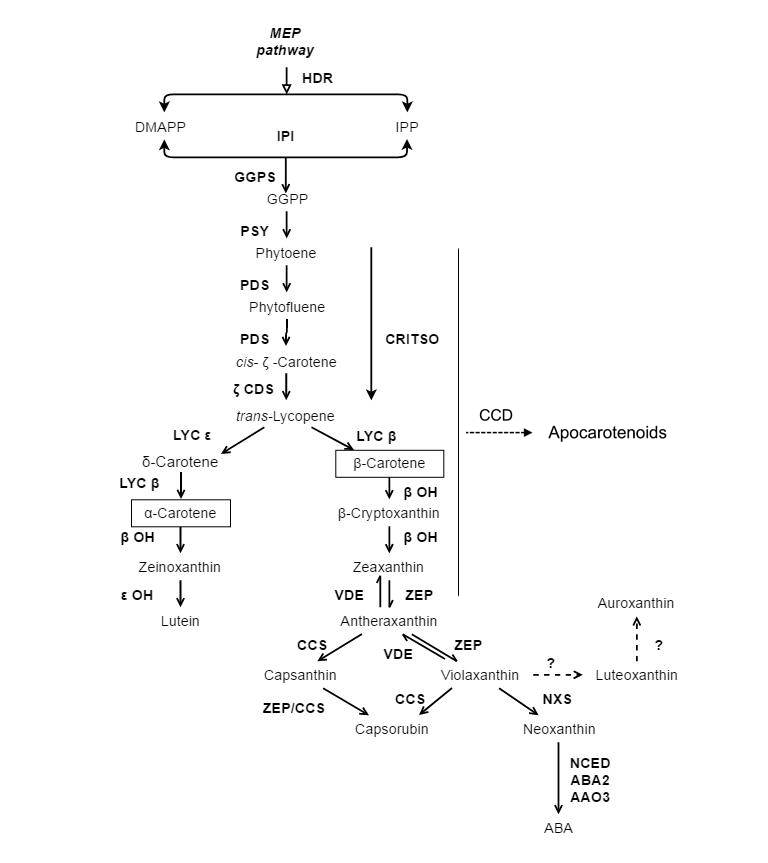
**

**S2 Fig**. **Carotenoid biosynthetic pathway in higher plants.** DMAPP: 3,3-dimethylpropylene pyrophosphoric acid; IPP: isopentenyl diphosphate; GGPP: Geranylgeranyl pyrophosphate ; ABA: Abscisic acid; Enzyme abbreviations: HDR: Hydroxymethylbutenyl -4- phosphate reductase; IPI: IPP isomerase; GGPS: Geranylgeranylpyrophosphate synthase; PSY: phytoene synthase; PDS: phytoenedesaturase; ζCDS: ζ-carotene desaturase; CRITSO: carotenoid isomerase; LCYβ: lycopeneβ-cyclase; LCYε:lycopene ε-cyclase; βOH: β-ring carotene hydroxylase;εOH: ε-ring carotene hydroxylase; VDE: violaxanthinde-epoxidase; ZEP: zeaxanthin epoxidase; CCD: carotenoid cleavage dioxygenases; CCS: Capsanthin synthase; NXS: neoxanthin synthase; NCED: Carotenoid cleavage enzymes ; ABA2: Short-chain dehydrogenase/redutase ; AAO3: Abscisic aldehyde oxidase
