# Supplementary material for: Chlorophyll, carotenoid and vitamin C metabolism regulation in Actinidia chinensis 'Hongyang' outer pericarp during fruit development
Source: PLoS One. 2018 Mar 26;13(3):e0194835. doi: 10.1371/journal.pone.0194835 (PMC5868826; doi:10.1371/journal.pone.0194835)
Supplement: S3 Fig — The pathway that uses GDP-L-gulose as a precursor has not been elucidated (Dotted line). AO, L-ascorbate oxidase; APX, L-ascorbate peroxidase; GPI, Glucose-6-phosphate isomerase; PMI, Phosphomannose isomerase; PMM, Phosphomannomutase; GMP, GDP-mannose pyrophosphorylase; GME, GDP-mannose-3,5-epimerase; GGP, GDP-L-galactose-1-phosphate phosphorylase; GPP, L-galactose-1-phosphate phosphatase; GalDH, L-galactose dehydrogenase; GLDH, L-galactono-1,4-lactone dehydrogenase; MDHAR: Monodehydroascorbate reductase; DHAR: Dehydroascorbate reductase. (DOC) [file pone.0194835.s003.doc]

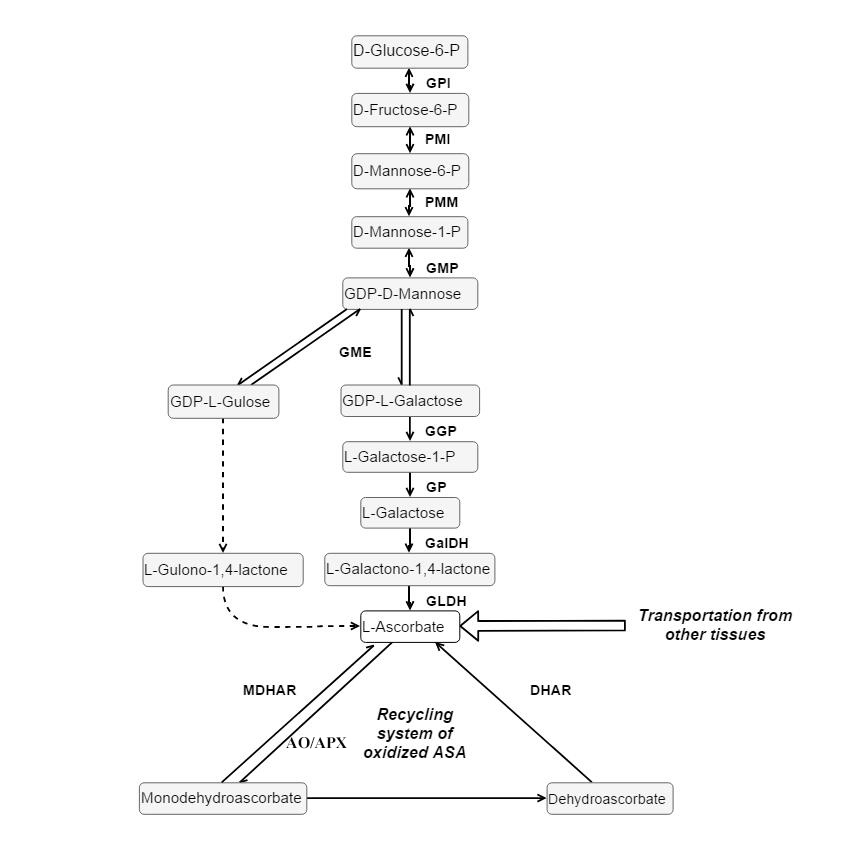


**S3 Fig**. **Possible schemes for ascorbate accumulation in fruits.** The pathway that uses GDP-L-guloseas a precursor has not been elucidated (Dotted line). AO, L-ascorbate oxidase; APX, L-ascorbate peroxidase; GPI, Glucose-6-phosphate isomerase; PMI, Phosphomannose isomerase; PMM, Phosphomannomutase; GMP, GDP-mannose pyrophosphorylase; GME, GDP-mannose-3,5-epimerase; GGP, GDP-L-galactose-1-phosphate phosphorylase; GPP, L-galactose-1-phosphate phosphatase; GalDH, L-galactose dehydrogenase; GLDH, L-galactono-1,4-lactone dehydrogenase; MDHAR: Monodehydroascorbate reductase; DHAR: Dehydroascorbate reductase.
