# Supplementary material for: Chlorophyll, carotenoid and vitamin C metabolism regulation in Actinidia chinensis 'Hongyang' outer pericarp during fruit development
Source: PLoS One. 2018 Mar 26;13(3):e0194835. doi: 10.1371/journal.pone.0194835 (PMC5868826; doi:10.1371/journal.pone.0194835)
Supplement: S4 Fig — Core tissue (pink), inner pericarp (green), outer pericarp (yellow), epidermis (blue). (DOC) [file pone.0194835.s004.doc]

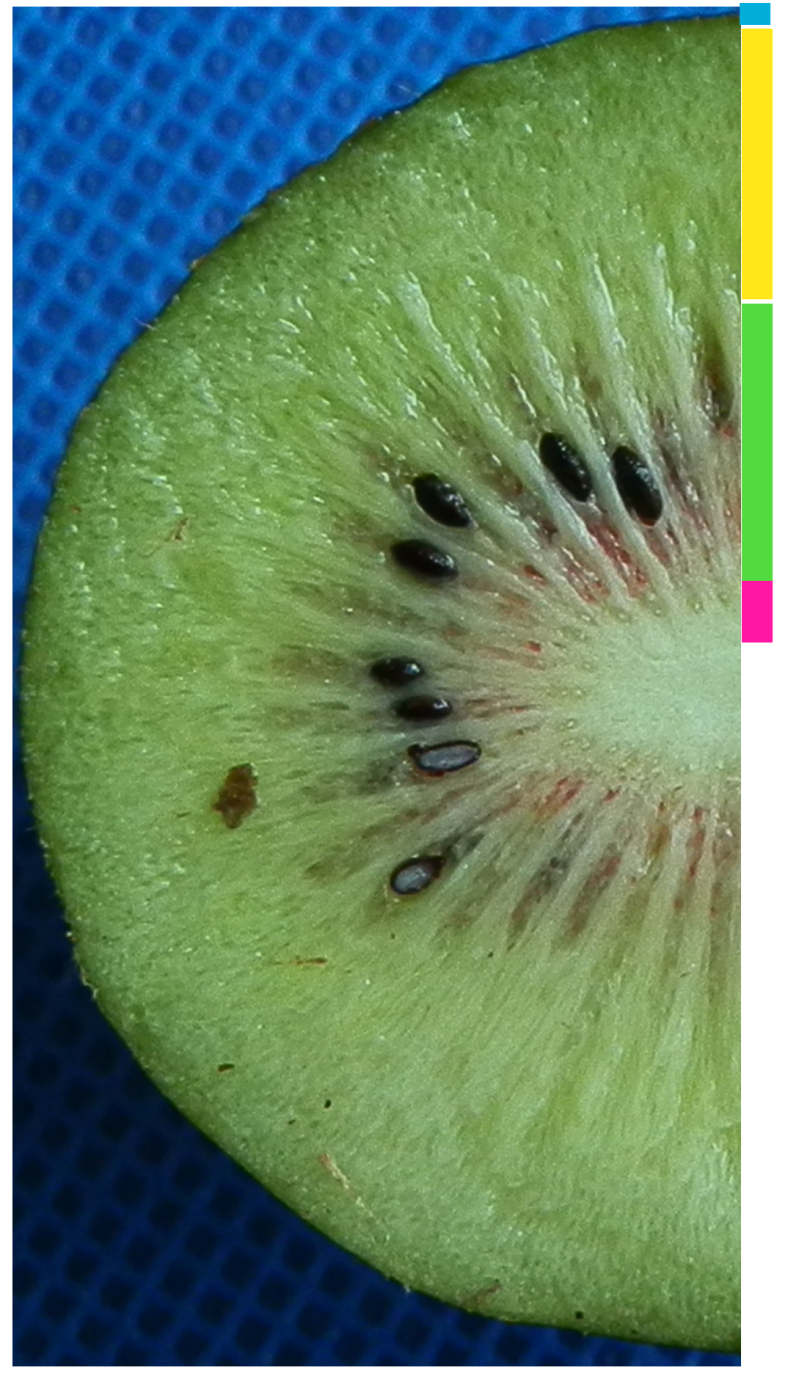


**S4 Fig**. **Tissue of *A. chinensis* var. *chinensis* ‘Hongyang’ fruit.Core tissue (pink), inner pericarp (green), outer pericarp (yellow), epidermis (blue).**
