# Supplementary material for: Chlorophyll, carotenoid and vitamin C metabolism regulation in Actinidia chinensis 'Hongyang' outer pericarp during fruit development
Source: PLoS One. 2018 Mar 26;13(3):e0194835. doi: 10.1371/journal.pone.0194835 (PMC5868826; doi:10.1371/journal.pone.0194835)
Supplement: S1 Table — (DOCX) [file pone.0194835.s005.docx]

**S1 Table Primers used in qRT-PCR analysis of *A. chinensis* var. *chinensis* ‘Hongyang’ outer pericarp.**

| **Gene name** | **Kiwifruit ID** | **Forward primer sequence 5’-3’** | **Reverse primer sequence 5’-3’** |
| --- | --- | --- | --- |
| **Chlorophyll biosynthesis and degradation related genes** | | | |
| *CAO1* | Achn353001 | TCCCGTTCCATCTTACCATCCC | TTATTGAGCCAAGGTGAAGAGGAC |
| *GluTR1* | Achn041311 | AGGTCAAACAAGTCGTCAAAGTCG | CGGTGATTGCGTGCTTAAACAG |
| *LHCB1* | Achn154321 | TTCGGGTTACAGCGGTTCAGG | ATCAGCGTGGTTATGGAGAAGGG |
| *LHCB2* | Achn152591 | ATGGATTGAGGTCCTAGTCATTGG | TCTTCTTCTCCTGGTCCGATGC |
| *RBCS1* | Achn311511 | TCAAGACTCTCAACTGGCTTCAAC | CATCAAACTCAAGGCAAGGGAACC |
| *CBR1* | Achn146001 | GTGAACCTGATGATGTGCGGAAG | TGTTCGTCCCAGCATTGTTTATCC |
| *CLH1* | Achn378091 | GCTCATCAGACACATCGCTTCC | CGTGGACTTGATCTCATCGGTTG |
| *CLH2* | Achn378101 | TGGTAATCGGGTCGGGTTTGG | AAGCCCTTGTTCTCATCATCTAGC |
| *CLS1* | Achn001951 | TTCAGTCACTTCCTGTGGCTTTTG | CCAAGAAGAGCAAGGGCGTTATAC |
| *PAO1* | Achn068721 | ACGCTCCGAATCTCTCCAACAG | CTTCCACTGCCTTCCCGACAC |
| *PAO2* | Achn212711 | TGAAGGAGGGTTCTGCTGATGTC | CGTTTGGATAGGATGGTTGATGGC |
| *PPH1* | Achn193491 | AGAGGATGGAAAGATCGTGAGAGG | CGAGCAACCAAAGGAGCCAATTC |
| *PPH2* | Achn193501 | GGAAGACGGTGTTGATCTGGTAAC | TAGCCGCCTCAATCCATCTGC |
| *PPH3* | Achn334341 | GGAAGACGGAAAGATAGCGAGAGG | TGCCATACCACGAACCAGGAC |
| *SGR1* | Achn361611 | CCAAGAACTTACACACTCACACAC | ACCAACCCTGTAACTGGGAATTG |
| **Carotenoid biosynthesis and degradation related genes** | | | |
| *ZISO1* | Achn248961 | GATGGTTGGGCAGGTGATTTGG | AATGACGCTTGTTCGGCTCTTC |
| *ZEP1* | Achn013171 | GCCTGCTGATATTGAGACTGTTGG | AGGTTGCTCGTTGTGAAATGCG |
| *ZDS1* | Achn274521 | TAGTTGGAGTGCCTGTGGTTACG | GCTTGCCTTAGTTGCCTTGATCG |
| *VDE1* | Achn368030 | TTGTGGATGAGTTCAACGAGTGTG | AGAGTAGGATCAGGGACAGGAAAG |
| *VDE2* | Achn368041 | GGTCCTGAACCACCCCTTGTAG | CCCACCTTCTCAACCTCCTCTTC |
| *PTOX1* | Achn150731 | GCTTGGTGGTTTGACCGCTTTC | GCTCCTCTCCTTGTGCCTTGATG |
| *PSY1* | Achn148841 | CGACCATCTACAATGCTGCTCTC | ACTCTTTCCACCCATCACTCACC |
| *PSY2* | Achn335751 | CGATGAGGCGGAGAAAGGAGTC | TCGTGAAGTTGTTGTAGTCGTTGG |
| *PDS1* | Achn199641 | TGTGAACCTTGCCGTCCCTTAC | TTTCCTGAAAGAACAGCACCTTCC |
| *NCED1* | Achn061851 | CAGAAAGCGGCGGCAATGG | GCGAAGTTCCCTGCGATTTGG |
| *NCED2* | Achn176351 | TGGTGTACGACGAGAAGAAGACG | CAGAGGTGGAAGCAGAAGCAATC |
| *LCYE1* | Achn061261 | CACAAGAAAGGAAACGCCAGAGAG | CCCGAGAAATCCCTGCCACATC |
| *LCYB1* | Achn198281 | CGAGCGTTGTGTGATTCCTATGG | GCTGCGGCTAGAGTCCTTGC |
| *LCYB2* | Achn347121 | GAGGCATTTGGGCATCAGAGTG | CTATGGCATCGGCTAGGACAGG |
| *CYP1* | Achn106371 | GTTATCGGCTCGTATTGCTTCGG | ACTAATCGCTCCCTTTGCCTCAG |
| *CYP2* | Achn318041 | CAGATTTTGCCTTGTTGCCTTTCG | CATTGCCAGTGCTACCGTTGAC |
| *CrtISO1* | Achn058301 | TTTGATAACCCTGGCATTGGAAGC | ACTCGGACGGAGAGATTGTTTGG |
| *CHY1* | Achn011691 | TCCTGCGATAGCCCTCCTCTC | GCCATCCCAAACACCGTTATTCC |
| *CCD1* | Achn378731 | AGAAGGCGGAGGAGGAGAGG | CTGGTGTGGCTGTGAAGAATCG |
| *CCD2* | Achn276731 | TGATGGGCGGGTGGTTTGTC | CTGTGTTGGTCACTATTGGATGCG |
| **AsA biosynthesis genes** | | | |
| *PGI1* | Achn087691 | AACCTGTTGAACCATTGACACTTG | TTGATGCTACGAGGCGAACC |
| *PGI2* | Achn197361 | CTCTTATCTGTGACACGGAGCAATG | GTGAGTAATCCAATAGCATCCCATCG |
| *PMI1* | Achn330131 | TTCACCGAACTCATGTCTGCTAG | CTTATCCGTCAACTGCCTCACC |
| *PMM1* | Achn302501 | TCACAGGCAGGTCCAGTCTC | AAGTGTAGGCAGCAGCAATCTC |
| *GMP1* | Achn055281 | GGTGGATGAGACCGCAACAATC | GGTTGAGTGCCAGCCGATAATG |
| *GME1* | Achn030021 | TGGAAAGGTGGAAGGGAGAAAGC | ATGAAGGTGAAAGATCGGGTTTGC |
| *GGP1* | Achn155031 | GAGGGTGAAAGAGGTTGTTGGTG | CGCAAGCAGTGACATCGTAGC |
| *GGP2* | Achn339231 | AACAGAGCAACGATAGCAAATCCC | GAGGCAAGCAGTCAAGAACACG |
| *GPP1* | Achn262331 | CTCAGAGTTCCTCGCCATTGC | GCCCTTATGCTCCACATGCTTG |
| *GPP2* | Achn341581 | ACTGAACCTTTGTGGGATTGC | CGCTGATGTCAAATTCTTTACCG |
| *GDH1* | Achn334011 | GCTTTGATTTCAGTGCCGAGAGAG | GGGAGTCCTGTAATACCAATAAACCG |
| *GalLDH1* | Achn136491 | TTAGGCTGGAGTGATGAGATTCTGG | TCATACTGGGCTTTGTTAAGGTTCC |
| *AO1* | Achn228031 | ACGACTTCTGGGTGTTGGGATAC | AGGCTCTATGTGGCAGTGGAATG |
| *AO2* | Achn230561 | AATGCCAACACAATGAATCCCAAC | CTCATAGCAGTCCAGCCGTAGG |
| *APX1* | Achn315041 | CTCCGCTTATGCTCCGTCTC | ACCTCCAGTCTTTGTCGTCAC |
| *APX2* | Achn289741 | GCTCTCATCTCCACCAAGAATTGC | TGACCTCAACTGCCACAACACC |
| *APX3* | Achn207061 | GAACTTCTGAATGAGTCGGAGGAG | ACAAGAGGACGATGGAGTGAACC |
| *DHAR1* | Achn224231 | ACCTTTGGTAACACCGCCTGAG | ATGCTTGCTCTGTTCCATTGCTG |
| *MDHAR1* | Achn005611 | GTGGTTGGTGGTGGTTACATTGG | TCGGCGAGGGAAGGAGTAAAC |
| *MDHAR2* | Achn132811 | AGTGGTGGTGGTTGGTGGTG | GGCGAGGGAAGGAGTAAACAATC |
| *MDHAR3* | Achn075231 | GGAGGAGGATACATCGGTCTTGAG | GCGTTAAACCCAACAGCCACAG |
| *MDHAR4* | Achn297231 | AGTCAGGAACCAGAACCAGAACC | CCGATGCTGCCACAATAACACC |
| *Actin* |  | TGCATGAGCGATCAAGTTTCAAG | TGTCCCATGTCTGGTTGATGACT |
