# Supplementary material for: Chlorophyll, carotenoid and vitamin C metabolism regulation in Actinidia chinensis 'Hongyang' outer pericarp during fruit development
Source: PLoS One. 2018 Mar 26;13(3):e0194835. doi: 10.1371/journal.pone.0194835 (PMC5868826; doi:10.1371/journal.pone.0194835)
Supplement: S2 Table — (DOC) [file pone.0194835.s006.doc]

S2 Table. Total carotenoid and total chlorophyll levels in *A. chinensis* var. *chinensis* ‘Hongyang’ (mg 100 g^-1^ fresh weight)

|  | **Chlorophyll a** | **Chlorophyll b** | **Total Chlorophyll** | **Total carotenoid** |
| --- | --- | --- | --- | --- |
| **100** | 3.40±0.14 c | 2.08±0.10 c | 5.48±0.27 c | 1.39±0.12 a |
| **120** | 2.48±0.13 b | 1.70±0.16 b | 4.18±0.25 b | 1.28±0.12 a |
| **141** | 2.41±0.13 b | 1.52±0.18 b | 3.93±0.23 b | 1.29±0.14 a |
| **148** | 1.63±0.12 a | 0.86±0.06 a | 2.49±0.13 a | 1.17±0.13 a |

The different small letters for number in a same column represent significant difference at 0.05 level.
