# Supplementary material for: Chlorophyll, carotenoid and vitamin C metabolism regulation in Actinidia chinensis 'Hongyang' outer pericarp during fruit development
Source: PLoS One. 2018 Mar 26;13(3):e0194835. doi: 10.1371/journal.pone.0194835 (PMC5868826; doi:10.1371/journal.pone.0194835)
Supplement: S3 Table — The different small letters for number in a same gene represent significant difference at 0.05 level. (DOCX) [file pone.0194835.s007.docx]

**S3 Table The relative expression (fold) of Chlorophyll biosynthesis and degradation related genes**

|  | **Genes name** | **DAA (fold change)** | | | |
| --- | --- | --- | --- | --- | --- |
|  |  | **100** | **120** | **141** | **148** |
| **Chlorophyll biosynthesis related genes** | *AcCAO1* | 5.00±1.13 b | 1.60±0.64 a | 1.00±0.32 a | 10.47±2.78 c |
|  | *AcGluTR1* | 2.96±0.82 a | 1.03+0.24 a | 1.00±0.39 a | 9.00±1.90 b |
|  | *AcLHCB1* | 10.67±0.59 c | 10.40±1.69 c | 4.49±0.34 b | 1.00±0.04 a |
|  | *AcLHCB2* | 40.11±8.79 c | 1.00±0.21 a | 1.98±0.42 a | 21.26±6.15 b |
|  | *AcRBCS1* | 1.99±0.27 a | 1.00±0.18 a | 1.68±0.21 a | 42.24±13.07 b |
|  | *AcCLS1* | 10.25±1.57 c | 9.63±1.47 c | 6.36±1.46 b | 1.00±0.18 a |
| **Chlorophyll degradation related genes** | *AcCLH1* | 11.02±2.06 b | 3.11±0.67 a | 1.98±0.30 a | 1.00±0.21 a |
|  | *AcCLH2* | 67.79±5.39 d | 32.40±4.28 c | 15.98±1.93 b | 1.00±0.08 a |
|  | *AcPAO2* | 3.24±0.58 b | 1.67+0.33 a | 1.00±0.24 a | 1.88±0.31 a |
|  | *AcPPH1* | 1.86±0.42 b | 2.77±0.25 c | 1.00±0.25 a | 1.61±0.10 b |
|  | *AcPPH2* | 2.14±0.43 b | 2.19±0.24 b | 1.00±0.26 a | 4.02±1.37 c |
|  | *AcPPH3* | 2.80±0.27 b | 1.00±0.12 a | 1.17±0.23 a | 6.33±1.53 c |
|  | *AcSGR2* | 5.79±1.63 b | 6.80±2.22 b | 5.88±1.65 b | 1.00±0.38 a |

The different small letters for number in a same gene represent significant difference at 0.05 level.
