# Supplementary material for: Chlorophyll, carotenoid and vitamin C metabolism regulation in Actinidia chinensis 'Hongyang' outer pericarp during fruit development
Source: PLoS One. 2018 Mar 26;13(3):e0194835. doi: 10.1371/journal.pone.0194835 (PMC5868826; doi:10.1371/journal.pone.0194835)
Supplement: S4 Table — **Correlation is significant at the P < 0.01 level (1-tailed), *correlation is significant at the P< 0.05 level (1-tailed). (DOC) [file pone.0194835.s008.doc]

**S4 Table. Pearson’s correlation (r) comparing relative gene expression during *A. chinensis* var. *chinensis* ‘Hongyang’ fruit development with total chlorophyll content**

| **Pearson's correlation(r)** | ***AcCAO1*** | ***AcGluTR1*** | ***AcLHCB1*** | ***AcLHCB2*** | ***AcCLH1*** | ***AcCLH2*** | ***AcRBCS1*** | ***AcCLS1*** | ***AcPAO1*** | ***AcPAO2*** | ***AcPPH1*** | ***AcPPH2*** | ***AcPPH3*** | ***AcCBR1*** | ***AcSGR1*** |
| --- | --- | --- | --- | --- | --- | --- | --- | --- | --- | --- | --- | --- | --- | --- | --- |
| **Total Chlorophyll** | -0.538  (0.231) | -0.676  (0.162) | 0.883  (0.059) | 0.380  (0.310) | 0.883  (0.058) | **0.959**  **(0.021*****)** | -0.826  (0.087) | **0.926**  **(0.037*****)** | 0.501  (0.249) | 0.586  (0.207) | 0.224  (0.388) | -0.603  (0.199) | -0.611  (0.195) | -0.769  (0.115) | 0.778  (0.111) |
| ***AcGluTR1*** | **0.984**  **(0.008******)** | - | - | - | - | - | - | - | - | - | - | - | - | - | - |
| ***AcLHCB1*** | -0.57  (0.215) | -0.693  (0.153) | - | - | - | - | - | - | - | - | - | - | - | - | - |
| ***AcLHCB2*** | 0.563  (0.218) | 0.418  (0.291) | 0.157  (0.421) | - | - | - | - | - | - | - | - | - | - | - | - |
| ***AcCLH1*** | -0.080  (0.460) | --0.251  (0.374) | -0.713  (0.143) | 0.766  (0.117) | - | - | - | - | - | - | - | - | - | - | - |
| ***AcCLH2*** | -0.300  (0.350) | -0.464  (0.268) | 0.874  (0.063) | 0.576  (0.212) | **0.963**  **(0.019*****)** | - | - | - | - | - | - | - | - | - | - |
| ***AcRBCS1*** | **0.919**  **(0.04*****)** | **0.974**  **(0.013*****)** | -0.798  (0.101) | 0.201  (0.400) | -0.466  (0.267) | -0.65  (0.175) | - | - | - | - | - | - | - | - | - |
| ***AcCLS1*** | -0.717  (0.141) | -0.826  (0.087) | **0.971**  **(0.014***) | 0.070  (0.465) | 0.688  (0.156) | 0.855  (0.072) | -0.915  (0.042) | - | - | - | - | - | - | - | - |
| ***AcPAO1*** | 0.459  (0.270) | 0.298  (0.351) | 0.332  (0.334) | **0.982**  **(0.009*****)** | 0.849  (0.076) | 0.698  (0.151) | 0.073  (0.464) | 0.232  (0.384) | - | - | - | - | - | - | - |
| ***AcPAO2*** | 0.356  (0.322) | 0.186  (0.407) | 0.495  (0.252) | **0.923**  **(0.038*****)** | 0.886  (0.057) | 0.784  (0.108) | -0.038  (0.481) | 0.376  (0.312) | **0.978**  **(0.011*****)** | - | - | - | - | - | - |
| ***AcPPH1*** | -0.119  (0.441) | -0.179  (0.411) | 0.643  (0.179) | -0.073  (0.464) | 0.171  (0.415) | 0.337  (0.331) | -0.196  (0.402) | 0.473  (0.263) | 0.086  (0.457) | 0.277  (0.361) | - | - | - | - | - |
| ***AcPPH2*** | **0.926**  **(0.037*****)** | **0.922**  **(0.039*****)** | -0.449  (0.275) | 0.362  (0.319) | 0.208  (0.396) | -0.351  (0.325) | 0.895  (0.052) | -0.646  (0.177) | 0.305  (0.348) | 0.268  (0.366) | 0.214  (0.393) | - | - | - | - |
| ***AcPPH3*** | **0.993**  **(0.003*****)** | **0.996**  **(0.002******)** | -0.661  (0.169) | 0.498  (0.251) | -0.168  (0.416) | -0.393  (0.304) | **0.950**  **(0.025*****)** | -0.791  (0.105) | 0.378  (0.311) | 0.259  (0.370) | -0.207  (0.397) | **0.905**  **(0.048*****)** | **-** | - | - |
| ***AcCBR1*** | **0.932**  **(0034*****)** | **0.967**  **(0.016*****)** | -0.669  (0.166) | 0.239  (0.381) | -0.397  (0.302) | -0.559  (0.221) | **0.980**  **(0.010*****)** | -0.825  (0.088) | 0.141  (0.430) | 0.067  (0.468) | -0.001  (0.499) | **0.964**  **(0.018*****)** | **0.942**  **(0.029*****)** | - | - |
| ***AcSGR1*** | **-0.932**  **(0.032*****)** | **-0.979**  **(0.010*****)** | 0.826  (0.087) | -0.284  (0.358) | 0.398  (0.301) | 0.609  (0.195) | **-0.988**  **(0.006******)** | **0.921**  **(0.039*****)** | -0.141  (0.430) | -0.006  (0.497) | 0.323  (0.339) | -0.849  (0.076) | **-0.967**  **(0.017*****)** | **-0.946**  **(0.027*****)** | - |

**correlation is significant at the *P* < 0.01 level (1-tailed), *correlation is significant at the *P* < 0.05 level (1-tailed)
