# Supplementary material for: Chlorophyll, carotenoid and vitamin C metabolism regulation in Actinidia chinensis 'Hongyang' outer pericarp during fruit development
Source: PLoS One. 2018 Mar 26;13(3):e0194835. doi: 10.1371/journal.pone.0194835 (PMC5868826; doi:10.1371/journal.pone.0194835)
Supplement: S5 Table — The different small letters for number in a same gene represent significant difference at 0.05 level. (DOCX) [file pone.0194835.s009.docx]

**S5 Table. The relative expression (fold) of Carotenoid biosynthesis and degradation related genes**

|  |  | **DAA** | | | |
| --- | --- | --- | --- | --- | --- |
|  | **Gene name** | **100** | **120** | **141** | **148** |
| **Carotenoid biosynthesis genes** | *AcPSY1* | 1.40±0.17 ab | 1.75±0.16 b | 1.00±0.09 a | 5.18±0.63 c |
|  | *AcPSY2* | 2.09±0.55 b | 1.00±0.34 a | 1.502±0.38 ab | 1.33±0.36 ab |
|  | *AcCritISO1* | 2.49±0.54 b | 1.36±0.26 a | 1.00±0.23 a | 2.35±0.41 b |
|  | *AcPTOX1* | 1.02±0.26 a | 1.00±0.33 a | 1.42±0.34 a | 3.29±0.85 b |
|  | *AcZDS1* | 2.00±0.34 c | 1.96±0.18 bc | 1.00±0.09 a | 1.56±0.17 b |
|  | *AcZISO1* | 2.83±0.58 c | 1.79±0.27 b | 2.03±0.41 b | 1.00±0.19 a |
|  | *AcPDS1* | 2.10±0.52 c | 1.64±0.44 ab | 1.82±0.47 ab | 1.00±0.34 a |
|  | *AcLCYB1* | 6.76±1.96 b | 3.10±0.93 a | 1.00±0.29 a | 1.13±0.35 a |
|  | *AcLCYB2* | 2.68±0.96 a | 1.00±0.15 a | 4.06±0.72 a | 37.45±4.32 b |
|  | *AcLCYE1* | 34.46±9.22 b | 26.23±7.45 b | 5.80±1.62 a | 1.00±0.35 a |
|  | *AcCYP1* | 2.12±0.42 b | 1.49±0.23 ab | 2.10±0.40 b | 1.00±0.20 a |
|  | *AcCYP2* | 10.79±2.45 c | 12.09±1.51 c | 5.59±0.95 b | 1.00±0.16 a |
|  | *AcCHY1* | 3.11±1.04 a | 1.38±0.38 a | 1.00±0.29 a | 63.12±14.61 b |
| **Carotenoid degradation related gene** | *AcNCED1* | 2.17±0.54 a | 2.62±0.62 a | 1.00±0.28 a | 133.71±31.37 b |
|  | *AcNCED2* | 26.17±4.80 c | 7.01±1.39 b | 1.00±0.25 a | 1.14±0.23 a |
|  | *AcZEP1* | 2.05±0.27 b | 1.00±0.20 a | 1.05±0.16 a | 1.17±0.22 a |
|  | *AcVDE1* | 89.45±13.58 d | 52.38±6.57 c | 18.01±2.79 b | 1.00±0.13 a |
|  | *AcVDE2* | 42.52±12.85 b | 43.23±10.87 b | 10.88±2.73 a | 1.00±0.35 a |
|  | *AcCCD1* | 18.68±2.37 c | 11.30±0.76 b | 9.08±1.21 b | 1.00±0.09 a |
|  | *AcCCD2* | 1.33±0.43 a | 1.00±0.06 a | 1.80±0.29 a | 3.12±0.65 b |

The different small letters for number in a same gene represent significant difference at 0.05 level.
