# Supplementary material for: Chlorophyll, carotenoid and vitamin C metabolism regulation in Actinidia chinensis 'Hongyang' outer pericarp during fruit development
Source: PLoS One. 2018 Mar 26;13(3):e0194835. doi: 10.1371/journal.pone.0194835 (PMC5868826; doi:10.1371/journal.pone.0194835)
Supplement: S6 Table — The different small letters for number in a same gene represent significant difference at 0.05 level. (DOCX) [file pone.0194835.s010.docx]

**S6 Table. The relative expression (fold) of AsA biosynthesis and recycling pathway genes**

|  |  | **DAA** | | | |
| --- | --- | --- | --- | --- | --- |
|  | **Genes name** | **30** | **58** | **141** | **148** |
| **AsA biosynthesis pathway genes** | *AcPGI1* | 1.00±0.13 a | 1.11±0.12 a | 1.01±0.13 a | 1.26±0.13 a |
|  | *AcPGI2* | 1.32±0.21 a | 1.14±0.13 a | 1.00±0.13 a | 1.17±0.16 a |
|  | *AcPMI1* | 4.63±0.53 c | 5.76±0.61 c | 2.42±0.61 b | 1.00±0.09 a |
|  | *AcPMM1* | 9.20±3.91 ab | 14.09±6.22 b | 5.78±3.01 ab | 1.00±0.35 a |
|  | *AcGMP1* | 10.09±1.89 c | 4.34±0.72 b | 1.37±0.26 a | 1.00**±**0.22 a |
|  | *AcGME1* | 55.29±9.04 c | 14.90±2.06 b | 5.98±0.82 ab | 1.00±0.18 a |
|  | *AcGGP1* | 15.98±3.37 b | 4.49±1.07 a | 2.56±0.52 a | 1.00±0.27 a |
|  | *AcGGP2* | 2.66±0.57 b | 1.08±0.20 a | 1.31±0.16 a | 1.00±0.03 a |
|  | *AcGPP1* | 5.44±1.36 b | 1.00±0.19 a | 1.11±0.20 a | 2.02±0.45 a |
|  | *AcGPP2* | 6.79±1.40 b | 1.11±0.21 a | 1.00±0.19 a | 2.08±0.37 a |
|  | *AcGDH1* | 712.63±188.70 b | 81.57±24.47 a | 54.08±11.68 a | 1.00±0.27 a |
|  | *AcGalLDH1* | 20.78±4.61 b | 4.06±0.74 a | 4.98±0.96 a | 1.00±0.18 a |
| **AsA recycling pathway genes** | *AcAO1* | 2320.15±923.32 b | 38.00±6.55 a | 2.13±0.23 a | 1.00±0.02 a |
|  | *AcAO2* | 22.55±3.92 b | 2.24±0.39 a | 3.40±0.23 a | 1.00±0.02 a |
|  | *AcAPX1* | 34.68±6.39 c | 13.86±2.38 b | 3.93±0.87 a | 1.00±0.23 a |
|  | *AcAPX2* | 1.88±0.29 b | 1.49±0.34 ab | 1.44±0.20 ab | 1.00±0.19 a |
|  | *AcAPX3* | 55.52±17.53 b | 23.43±7.80 a | 1.00±0.23 a | 12.76±4.20 a |
|  | *AcDHAR1* | 3.03±0.33 b | 2.47±0.33 b | 2.87±0.31 b | 1.00±0.13 a |
|  | *AcMDHAR1* | 5.70±0.46 d | 3.18±0.33 b | 4.51±0.48 c | 1.00±0.04 a |
|  | *AcMDHAR2* | 3.82±0.61 c | 2.20±0.20 b | 3.90±0.40 c | 1.00±0.12 a |
|  | *AcMDHAR3* | 1.40±0.35 a | 1.00±0.31 a | 1.01±0.25 a | 1.68±0.37 a |
|  | *AcMDHAR4* | 1.39±0.21 a | 2.16±0.43 b | 2.09±0.21 b | 1.00±0.08 a |

The different small letters for number in a same gene represent significant difference at 0.05 level.
