# Supplementary material for: Chlorophyll, carotenoid and vitamin C metabolism regulation in Actinidia chinensis 'Hongyang' outer pericarp during fruit development
Source: PLoS One. 2018 Mar 26;13(3):e0194835. doi: 10.1371/journal.pone.0194835 (PMC5868826; doi:10.1371/journal.pone.0194835)
Supplement: S7 Table — **Correlation is significant at the P < 0.01 level (1-tailed), *correlation is significant at the P< 0.05 level (1-tailed). (DOC) [file pone.0194835.s011.doc]

**S7 Table. Pearson’s correlation (r) comparing relative gene expression during *A. chinensis* var. *chinensis* ‘Hongyang’ fruit development with AsA content**

| Pearson's  correlation(r) | ***PGI1*** | ***PGI2*** | ***PMI1*** | ***PMM1*** | ***GMP1*** | ***GME1*** | ***GGP1*** | ***GGP2*** | ***GPP1*** | ***GPP2*** | ***GDH1*** | ***GalLDH1*** | ***AO1*** | ***AO2*** | ***APX1*** | ***APX2*** | ***APX3*** | ***DHAR1*** | ***MDHAR1*** | ***MDHAR2*** | ***MDHAR3*** | ***MDHAR4*** |
| --- | --- | --- | --- | --- | --- | --- | --- | --- | --- | --- | --- | --- | --- | --- | --- | --- | --- | --- | --- | --- | --- | --- |
| **AsA content** | -0.497  (0.252) | 0.875  (0.063) | 0.631  (0.185) | 0.481  (0.260) | **0.997**  (**0.002****) | **0.985**  (**0.007****) | **0.979**  (**0.010***) | **0.907**  **(0.047*****)** | 0.883  (0.058) | **0.909**  **(0.046*****)** | **0.960**  (**0.020***) | 0.936  (0.032) | **0.947**  **(0.027*****)** | 0.934  (0.033*****) | **0.992**  (**0.004****) | 0.842  (0.079) | **0.987**  (**0.007****) | 0.521  (0.239) | 0.658  (0.171) | 0.427  (0.286) | 0.113  (0.444) | -0.167  (0.416) |
| ***PGI2*** | -0.027  (0.486) | - | - | - | - | - | - | - | - | - | - | - | - | - | - | - | - | - | - | - | - | - |
| ***PMI1*** | -0.522  (0.239) | 0.355  (0.323) | - | - | - | - | - | - | - | - | - | - | - | - | - | - | - | - | - | - | - | - |
| ***PMM1*** | -0.510  (0.245) | 0.180  (0.410) | **0.983**  (**0.009******)** | - | - | - | - | - | - | - | - | - | - | - | - | - | - | - | - | - | - | - |
| ***GMP1*** | -0.561  (0.219) | 0.833  (0.083) | 0.670  (0.165) | 0.529  (0.235) | - | - | - | - | - | - | - | - | - | - | - | - | - | - | - | - | - | - |
| ***GME1*** | -0.606  (0.197) | 0.812  (0.094) | 0.573  (0.214) | 0.425  (0.287) | **0.988**  (**0.006****) | - | - | - | - | - | - | - | - | - | - | - | - | - | - | - | - | - |
| ***GGP1*** | -0.614  (0.193) | 0.804  (0.098) | 0.551  (0.225) | 0.403  (0.298) | **0.983**  (**0.009****) | **0.999**  **(0.009****) | - | - | - | - | - | - | - | - | - | - | - | - | - | - | - | - |
| ***GGP2*** | -0.648  (0.176) | 0.727  (0.136) | 0.365  (0.317) | 0.217  (0.392) | **0.910**  (**0.045***) | **0.962**  **(0.019***) | **0.971**  **(0.015***) | - | - | - | - | - | - | - | - | - | - | - | - | - | - | - |
| ***GPP1*** | -0.347  (0.327) | 0.875  (0.062) | 0.195  (0.103) | 0.017  (0.491) | 0.858  (0.071) | **0.906**  **(0.047***) | **0.913**  **(0.047***) | **0.939**  **(0.031***) | - | - | - | - | - | - | - | - | - | - | - | - | - | - |
| ***GPP2*** | -0.372  (0.314) | 0.855  (0.057) | 0.251  (0.375) | 0.075  (0.463) | 0.886  (0.057) | **0.928**  **(0.036***) | **0.933**  **(0.033***) | **0.948**  **(0.026***) | **0.998**  **(0.001****) | - | - | - | - | - | - | - | - | - | - | - | - | - |
| ***GDH1*** | -0.585  (0.207) | 0.810  (0.095) | 0.450  (0.275) | 0.294  (0.353) | **0.958**  **(0.021***) | **0.990**  **(0.005****) | **0.993**  **(0.003****) | **0.988**  **(0.006****) | **0.949**  **(0.025***) | **0.963**  **(0.018***) | - | - | - | - | - | - | - | - | - | - | - | - |
| ***GalLDH1*** | -0.676  (0.162) | 0.734  (0.133) | 0.459  (0.270) | 0.316  (0.342) | **0.943**  **(0.029***) | **0.982**  **(0.009****) | **0.987**  **(0.006****) | **0.995**  **(0.003****) | **0.918**  **(0.041***) | **0.932**  **(0.034***) | **0.993**  **(0.004****) | - | - | - | - | - | - | - | - | - | - | - |
| ***AO1*** | -0.527  (0.237) | 0.834  (0.083) | 0.380  (0.310) | 0.217  (0.392) | **0.938**  **(0.031***) | **0.976**  **(0.012***) | **0.980**  **(0.010***) | **0.985**  **(0.007****) | **0.973**  **(0.013***) | **0.983**  **(0.009****) | **0.996**  **(0.002****) | **0.982**  **(0.009****) | - | - | - | - | - | - | - | - | - | - |
| ***AO2*** | -0.603  (0.198) | 0.779  (0.111) | 0.392  (0.304) | 0.238  (0.381) | **0.933**  **(0.034***) | **0.976**  **(0.012***) | **0.983**  **(0.009****) | **0.997**  **(0.002****) | **0.964**  **(0.023***) | **0.964**  **(0.018***) | **0.997**  **(0.002****) | **0.995**  **(0.003****) | **0.995**  **(0.002****) | - | - | - | - | - | - | - | - | - |
| ***APX1*** | -0.596  (0.202) | 0.809  (0.095) | 0.677  (0.162) | 0.539  (0.231) | **0.999**  **(0.009****) | **0.990**  **(0.005**** | **0.985**  **(0.007****) | **0.916**  **(0.042***) | 0.850  **(**0.075) | 0.879  **(**0.061) | **0.960**  **(0.020***) | **0.950**  **(0.025***) | **0.938**  **(0.031***) | **0.936**  **(0.032***) | - | - | - | - | - | - | - | - |
| ***APX2*** | -0.878  (0.061) | 0.477  (0.262) | 0.735  (0.133) | 0.655  (0.173) | 0.883  **(**0.059) | 0.889  **(**0.056) | 0.888  **(**0.056) | 0.848  **(**0.076) | 0.640  **(**0.180) | 0.675  **(**0.163) | 0.848  **(**0.076) | 0.890  **(**0.055) | 0.799  **(**0.101) | 0.838  **(**0.081) | **0.902**  **(0.049***) | - | - | - | - | - | - | - |
| ***APX3*** | -0.349  (0.326) | **0.941**  (**0.030***) | 0.579  (0.211) | 0.418  (0.291) | **0.971**  **(0.015***) | **0.951**  **(0.025***) | **0.943**  **(0.028*****)** | 0.860  **(**0.07) | 0.892  **(**0.054) | **0.914**  **(0.043*****)** | **0.928**  **(0.036*****)** | 0.885  **(**0.058) | **0.825**  **(0.037*****)** | 0.897  **(**0.051) | 0.960  **(**0.020) | 0.744  **(**0.128) | - | - | - | - | - | - |
| ***DHAR1*** | **-0.987**  (**0.007****) | 0.044  (0.478) | 0.648  (0.176) | 0.643  (0.178) | 0.588  **(**0.206) | 0.607  **(**0.196) | 0.611  **(**0.194) | 0.607  **(**0.196) | 0.298  **(**0.351) | 0.332  **(**0.334) | 0.565  **(**0.218) | 0.650  **(**0.175) | 0.497  **(**0.251) | 0.570  **(**0.215) | 0.622  **(**0.189) | 0.899  **(**0.050) | 0.376  **(**0.312) | - | - | - | - | - |
| ***MDHAR1*** | **-0.977**  (**0.012***) | 0.233  (0.383) | 0.535  (0.232) | 0.484  (0.258) | 0.710  **(**0.145) | 0.757  **(**0.122) | 0.765  **(**0.117) | 0.796  **(**0.102) | 0.538  **(**0.231) | 0.562  **(**0.219) | 0.745  **(**0.127) | 0.818  **(**0.091) | 0.697  **(**0.152) | 0.760  **(**0.120) | 0.739  **(**0.130) | **0.942**  **(0.029*****)** | 0.527  **(**0.236) | **0.958**  **(0.021***) | - | - | - | - |
| ***MDHAR2*** | **-0.980**  (**0.010***) | -0.020  (0.490) | 0.344  (0.328) | 0.329  (0.336) | 0.487  **(**0.257) | 0.559  **(**0.220) | 0.574  **(**0.213) | 0.655  **(**0.172) | 0.369  **(**0.316) | 0.383  **(**0.308) | 0.568  **(**0.216) | 0.662  **(**0.169) | 0.521  **(**0.240) | 0.601  **(**0.200) | 0.523  **(**0.238) | 0.809  **(**0.096) | 0.278  **(**0.361) | **0.934**  **(0.033***) | **0.959**  **(0.021***) | - | - | - |
| ***MDHAR3*** | 0.621  (0.190) | 0.535  (0.232) | -0.571  (0.215) | -0.694  (0.153) | 0.033  **(**0.484) | 0.075  **(**0.463) | 0.081  **(**0.459) | 0.141  **(**0.429) | 0.464  **(**0.268) | 0.425  **(**0.288) | 0.164  **(**0.418) | 0.073  **(**0.464) | 0.251  **(**0.375) | 0.177  **(**0.412) | 0.001  **(**0.500) | -0.383  **(**0.309) | 0.242  **(**0.379) | -0.693  **(**0.153) | -0.460  **(**0.270) | -0.526  **(**0.237) | - | - |
| ***MDHAR4*** | -0.556  (0.222) | -0.567  (0.216) | 0.556  (0.222) | 0.687  (0.156) | -0.089  **(**0.455) | -0.138  **(**0.431) | -0.146  **(**0.427) | -0.216  **(**0.392) | -0.525  **(**0.238) | -0.486  **(**0.257) | -0.233  **(**0.383) | -0.145  **(**0.427) | -0.317  **(**0.341) | -0.247  **(**0.377) | -0.058  **(**0.471) | 0.317  **(**0.341) | -0.289  **(**0.356) | 0.635  **(**0.182) | 0.389  **(**0.306) | 0.458  **(**0.271) | **-0.997**  **(0.002****) | - |

**correlation is significant at the *P* < 0.01 level (1-tailed), *correlation is significant at the *P* < 0.05 level (1-tailed)
